# From defense to offense: antimicrobial peptides as promising therapeutics for cancer

**DOI:** 10.3389/fonc.2024.1463088

**Published:** 2024-10-09

**Authors:** Hadi Zare-Zardini, Elham Saberian, Andrej Jenča, Razieh Ghanipour-Meybodi, Andrej Jenča, Adriána Petrášová, Janka Jenčová

**Affiliations:** ^1^ Department of Biomedical Engineering, Meybod University, Meybod, Iran; ^2^ Klinika of Stomatology and Maxillofacial Surgery Akadémia Košice Bacikova, Pavel Jozef Šafárik University (UPJS LF), Kosice, Slovakia

**Keywords:** antimicrobial peptides, cancer therapy, immunomodulation, cytotoxicity, tumor

## Abstract

Antimicrobial peptides (AMPs), naturally occurring components of innate immunity, are emerging as a promising new class of anticancer agents. This review explores the potential of AMPs as a novel class of anticancer agents. AMPs, naturally occurring peptides with broad-spectrum antimicrobial activity, exhibit several characteristics that make them attractive candidates for cancer therapy, including selectivity for cancer cells, broad-spectrum activity, and immunomodulatory effects. Analysis of a dataset of AMPs with anticancer activity reveals that their effectiveness is influenced by various structural properties, including net charge, length, Boman index, and hydrophobicity. These properties contribute to their ability to target and disrupt cancer cell membranes, interfere with intracellular processes, and modulate the immune response. The review highlights the promising potential of AMPs as a new frontier in cancer treatment, offering hope for more effective and less toxic therapies. AMPs demonstrate promising potential in cancer therapy through multiple mechanisms, including direct cytotoxicity, immune response modulation, and targeting of the tumor microenvironment, as evidenced by extensive preclinical studies in animal models showing tumor regression, metastasis inhibition, and improved survival rates. AMPs show significant potential as cancer therapeutics through their direct cytotoxicity, immune response modulation, and tumor microenvironment targeting, with promising results from preclinical studies and early-phase clinical trials. Future research should focus on optimizing AMP properties, developing novel delivery strategies, and exploring synergistic combination therapies to fully realize their potential as effective cancer treatments, while addressing challenges related to stability, delivery, and potential toxicity.

## 1 Introduction

Cancer, a leading cause of death worldwide, continues to pose a significant challenge to global health (1). Despite advancements in conventional therapies such as surgery, chemotherapy, and radiotherapy, the treatment of cancer remains fraught with limitations (2–4). These modalities often come with debilitating side effects, lack selectivity for cancer cells, and struggle to combat drug resistance, leading to a persistent need for innovative therapeutic approaches (5–7). In this context, antimicrobial peptides (AMPs) emerge as a promising frontier in cancer therapy, offering a novel and multifaceted strategy to combat this formidable disease (8, 9). AMPs are naturally occurring, short-chain peptides found throughout the biological world, playing a crucial role in innate immunity (10). They act as the first line of defense against invading pathogens, exhibiting broad-spectrum antimicrobial activity against bacteria, fungi, viruses, and parasites (11, 12). Their diverse range of structures, with variations in amino acid sequence, length, and charge distribution, contributes to their potent antimicrobial effects (13). AMPs exert their antimicrobial activity through various mechanisms, often targeting the cell membrane of pathogens, disrupting its integrity, and leading to cell lysis (14). While their primary function lies in host defense, recent research has unveiled the remarkable potential of AMPs in cancer therapy. This emerging field holds significant promise due to the unique properties of AMPs, offering distinct advantages over conventional therapies (15). AMPs exhibit a remarkable ability to selectively target and kill cancer cells while sparing healthy cells. This selectivity stems from the inherent differences between the cell membranes of cancer cells and normal cells. AMPs preferentially bind to and disrupt the altered membranes of cancer cells, leading to their selective destruction (16). Furthermore, AMPs often exhibit broad-spectrum activity, effectively targeting a wide range of cancer types. This makes them particularly attractive for treating complex or multi-drug resistant cancers, where conventional therapies may struggle to achieve efficacy (17, 18). AMPs can also modulate the immune response, stimulating the immune system to recognize and eliminate cancer cells. They can directly activate immune cells, enhance cytokine production, and promote antigen presentation, leading to a robust anti-tumor immune response. Beyond their direct cytotoxic and immunomodulatory effects, AMPs can also disrupt the tumor microenvironment, targeting processes that contribute to tumor growth and metastasis (19, 20). They can inhibit angiogenesis, the formation of new blood vessels that supply tumors with nutrients and oxygen, effectively starving them. AMPs can also suppress metastasis by interfering with cell adhesion, migration, and extravasation, preventing the spread of cancer cells to distant sites (21). The unique properties of AMPs, including their selectivity, broad-spectrum activity, immunomodulatory effects, and ability to target the tumor microenvironment, make them a highly promising frontier in cancer therapy. Preclinical studies in animal models have demonstrated their efficacy against various cancer types, including solid tumors and hematologic malignancies (22, 23). The encouraging results from these studies have led to the initiation of clinical trials in humans, exploring the safety and efficacy of AMPs for cancer treatment, especially for reduction of side effects of chemotherapy (24). While AMPs hold significant promise, several challenges remain in translating them into effective cancer therapies. These challenges include improving their stability, optimizing delivery strategies, and addressing potential toxicity. Overcoming these hurdles requires focused research and development efforts, including the development of novel AMP candidates, the optimization of delivery systems, and the exploration of synergistic combination therapies. With continued research and development, AMPs have the potential to revolutionize cancer treatment, offering patients new hope and improving outcomes. This review delves into the mechanisms of action of AMPs in cancer, explores preclinical studies and clinical trials, and discusses the challenges and future directions for this exciting field.

## 2 The global burden of cancer

Cancer represents a major global health challenge, with rising incidence and mortality rates worldwide. It is estimated that by 2030, the world will see approximately 26 million new cancer diagnoses and 17 million cancer-related fatalities annually (25). This alarming trend underscores the urgent need for new and effective cancer therapies. While significant progress has been made in cancer treatment, current modalities often face limitations. Surgery, while effective for localized tumors, may not be feasible for advanced or metastatic cancers. Chemotherapy, a mainstay of cancer treatment, often suffers from severe side effects, including nausea, hair loss, and immunosuppression. Moreover, chemotherapy can be ineffective against certain types of cancer, and resistance to these drugs is a growing concern. Radiotherapy, though effective in targeting specific areas, can also damage healthy tissues and is not always suitable for all types of cancer. These limitations highlight the need for alternative therapeutic approaches that are more targeted, have fewer side effects, and are effective against a broader range of cancers (25, 26).

## 3 Antimicrobial peptides: a promising class of therapeutics

AMPs are naturally occurring, short-chain peptides with broad-spectrum antimicrobial activity. Found throughout the biological world, from bacteria to humans, AMPs play a crucial role in innate immunity, forming the first line of defense against invading pathogens. These peptides are characterized by their diverse range of structures, with variations in amino acid sequence, length, and charge distribution. This structural diversity contributes to their broad-spectrum activity, enabling them to target a wide range of microbes, including bacteria, fungi, viruses, and parasites. While AMPs are well-known for their antimicrobial properties, recent research has also highlighted their potential as therapeutic agents for cancer treatment. The anticancer activity of AMPs has been described in the literature for some time, suggesting that this is not a novel activity. AMPs can exert their anticancer effects through various mechanisms, including direct cytotoxicity towards cancer cells, modulation of the immune response, and induction of apoptosis. Their ability to selectively target cancer cells while sparing normal cells makes them attractive candidates for cancer therapy. The diverse nature of AMPs, with their unique structures and mechanisms of action, presents a vast reservoir of potential therapeutic agents for various applications, including cancer treatment. Ongoing research continues to explore and optimize the use of AMPs as novel anticancer agents, with the aim of developing effective and safe therapies for patients (27–29).

## 4 AMPs in cancer therapy: a promising frontier

The exploration of AMPs in cancer therapy represents a promising frontier in the field of oncology (30). Traditionally recognized for their role in defending against microbial infections, AMPs are now being investigated for their potential anticancer properties. This emerging area of research is driven by the unique characteristics of AMPs that may offer significant advantages over conventional cancer therapies (31). AMPs exhibit several features that make them attractive candidates for cancer treatment. One of the key advantages is their selectivity. Unlike traditional chemotherapeutics, which often lack specificity and can cause severe side effects by damaging healthy cells, AMPs have the potential to target cancer cells more selectively. This selectivity can be attributed to differences in the membrane composition and physiology of cancer cells compared to normal cells (32). For instance, cancer cell membranes often have altered lipid compositions and higher levels of specific receptors, which can be exploited by certain AMPs to enhance their binding and internalization. Another significant advantage of AMPs is their broad-spectrum activity. While conventional cancer therapies are typically designed to target specific molecular pathways or cellular processes, AMPs can exert their effects through multiple mechanisms (33–35). This includes direct cytotoxicity through membrane disruption, induction of apoptosis, and modulation of the immune response. The ability of AMPs to engage multiple pathways simultaneously can enhance their efficacy and potentially overcome resistance mechanisms that cancer cells may develop against single-target therapies. Furthermore, AMPs have shown potential for synergistic effects when combined with other therapeutic modalities. For example, the immunomodulatory properties of some AMPs can enhance the body’s natural antitumor immune response, making them ideal candidates for combination therapies with immunotherapies. Additionally, the direct cytotoxic effects of AMPs can complement traditional chemotherapy and radiotherapy, potentially improving treatment outcomes by targeting both the tumor and its microenvironment. In summary, the emerging potential of AMPs in cancer therapy is supported by their selectivity, broad-spectrum activity, and ability to synergize with other treatments. These advantages position AMPs as a promising frontier in the development of novel anticancer agents, offering hope for more effective and less toxic therapies in the fight against cancer (35–37). All reported AMPs with anticancer activity were summarized in **Supplementary Table 1**. This table summarizes the key characteristics and anti-cancer activities of antimicrobial peptides identified through a comprehensive review of published literature. The table includes information on peptide source, sequence, structure, charge, hydrophobicity, and Boman index.

**Figure 1:**
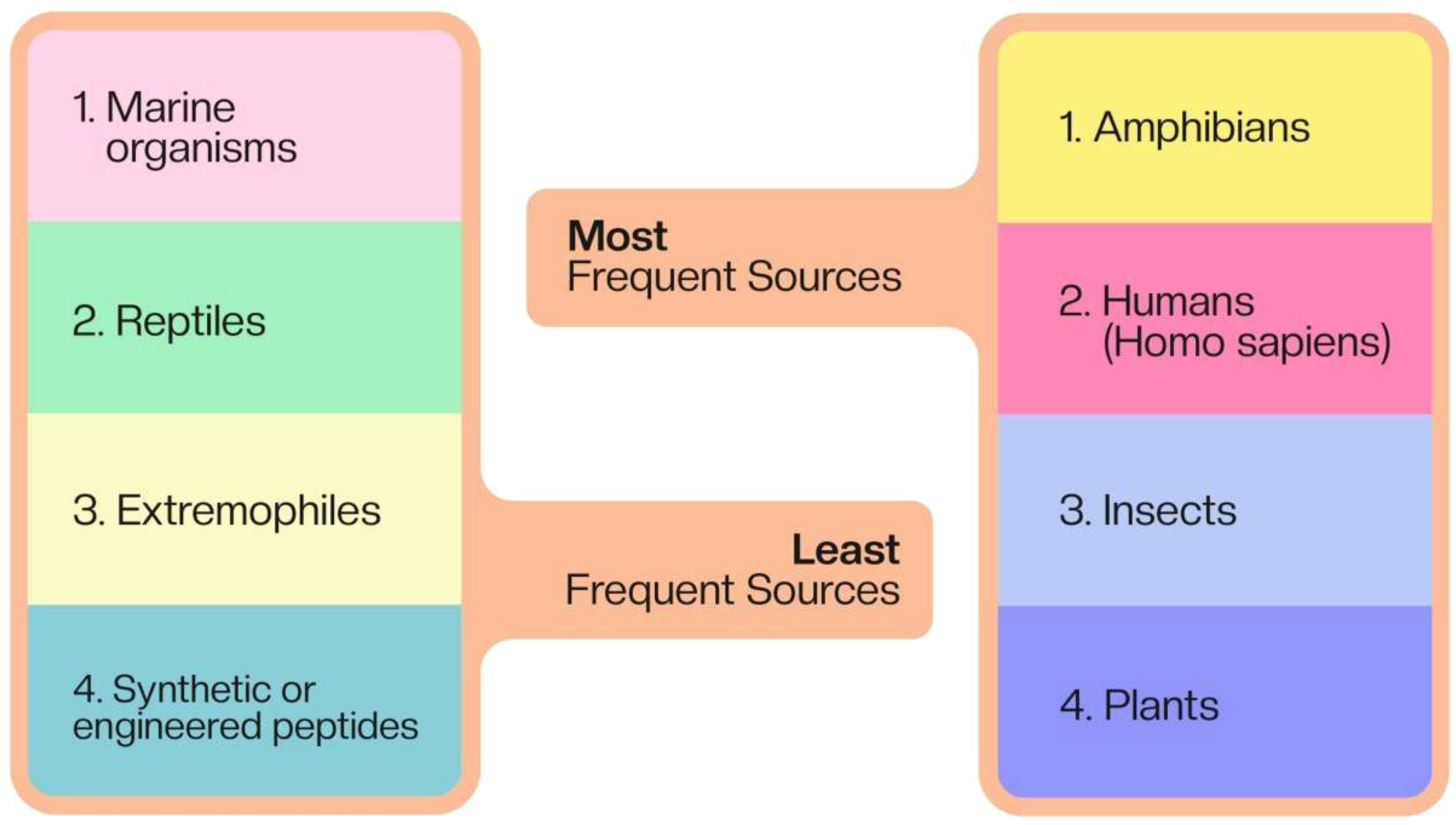
The sources of antimicrobial peptides with anticancer effects.

The table provided offers a comprehensive overview of the sources of AMPs with anticancer effects. This diverse array of sources highlights the ubiquitous nature of these peptides and their potential as a rich resource for novel anticancer therapeutics (**Figure 1**).

### 4.1 Most frequent sources

1. Amphibians: Frogs and toads are the most abundant sources of AMPs in this dataset. Species from genera such as Litoria, Rana, Bombina, and Phyllomedusa are particularly well-represented. This prevalence is likely due to the rich diversity of peptides in amphibian skin secretions, which serve as a defense mechanism against pathogens in their environment.
2. Humans (Homo sapiens): A significant number of AMPs are derived from various human tissues and fluids, including neutrophils, skin, saliva, and colonic mucosa. This highlights the potential for developing endogenous human peptides as therapeutics, potentially reducing immunogenicity issues.
3. Insects: Bees, wasps, and various fly species contribute a notable number of AMPs. Venom-derived peptides from these insects often exhibit potent antimicrobial and anticancer properties.
4. Plants: Several plant species, particularly from genera like Viola, Clitoria, and Viscum, are sources of AMPs. This demonstrates the potential of plant-derived peptides in cancer therapy.

### 4.2 Least frequent sources

1. Marine organisms: While present, AMPs from marine sources such as fish and marine invertebrates are less represented compared to terrestrial sources.
2. Reptiles: Only a few entries are from reptilian sources, such as crocodiles and snakes.
3. Extremophiles: There are limited entries from extremophile organisms, such as alkalophilic bacteria.
4. Synthetic or engineered peptides: While present, purely synthetic or engineered peptides are less common than naturally derived ones.

This distribution reflects both the natural abundance of AMPs in certain organisms and the research focus in the field. Amphibians, particularly frogs, have been extensively studied due to their rich repertoire of bioactive peptides. The significant presence of human-derived AMPs suggests a growing interest in developing endogenous peptides as therapeutics.

The diversity of sources underscores the vast potential for discovering novel anticancer peptides across different kingdoms of life. It also highlights opportunities for exploring less-studied sources, such as marine organisms and extremophiles, which might yield unique peptides with advantageous properties.

Future research could benefit from a more balanced approach, investigating underrepresented sources while continuing to explore the rich diversity of well-established sources like amphibians and insects. Additionally, the development of synthetic and engineered peptides based on natural templates offers promising avenues for optimizing anticancer efficacy and reducing potential side effects.

In conclusion, this dataset demonstrates the wide distribution of anticancer AMPs in nature and emphasizes the importance of biodiversity in drug discovery efforts. It also underscores the potential for developing novel anticancer therapies from both natural and engineered peptide sources.

**Figure 2:**
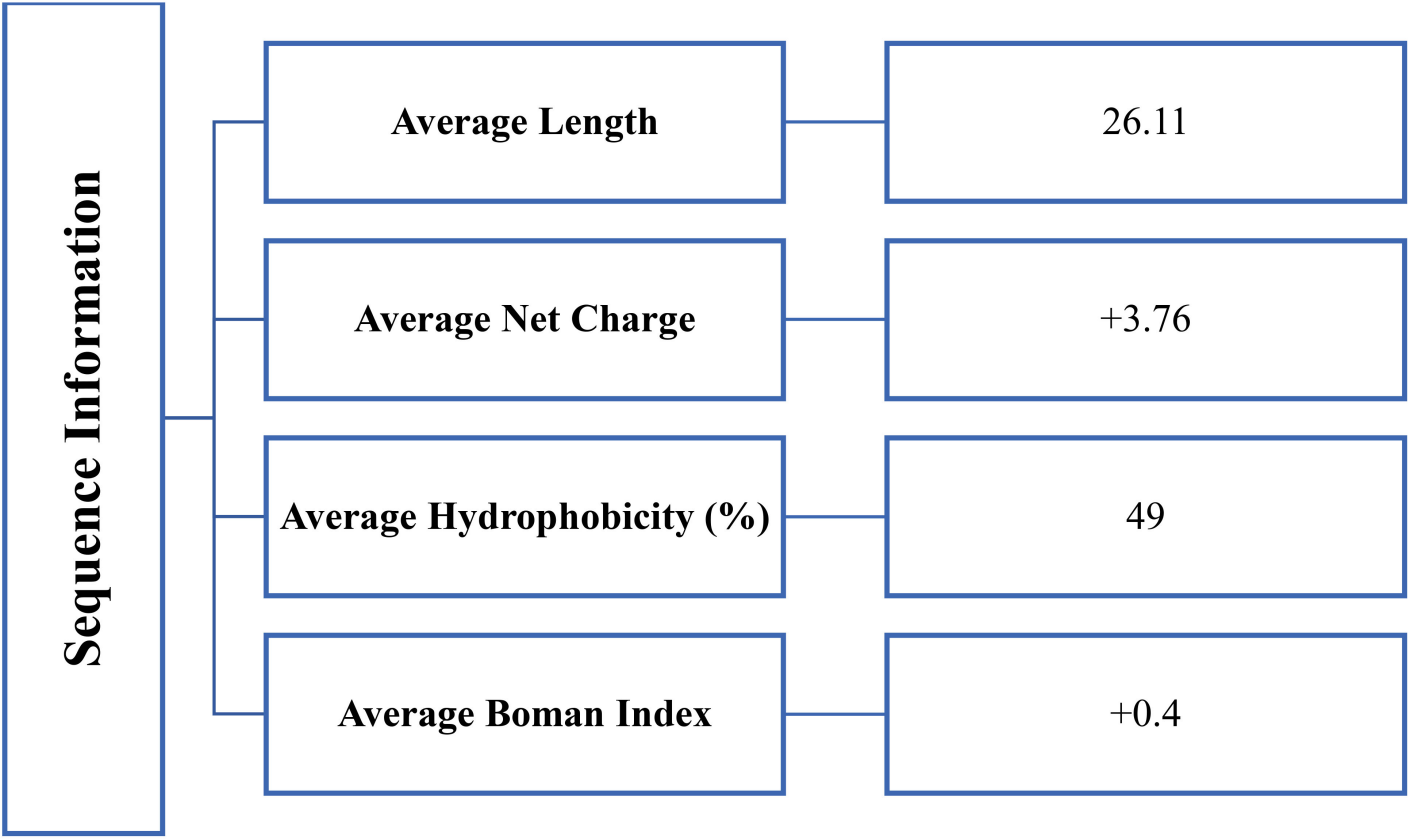
Physicochemical properties based upon sequence information of reported antimicrobial peptides with anticancer activity.

The sequence information of reported anticancer peptides was shown in **Figure 2**. The data presented on antimicrobial peptides with anti-cancer effects provides valuable insights into how specific structural characteristics influence their anticancer activities. The impact of net charge, peptide length, Boman index, and hydrophobicity on the anti-cancer properties of these peptides can be considered as below:

Net Charge: The net charge of peptides plays a crucial role in their anti-cancer activities. Positively charged peptides are generally more effective against cancer cells due to:

- Enhanced electrostatic interaction with the negatively charged cancer cell membranes.
- Increased ability to disrupt cancer cell membranes, leading to cell death.
- Potential to target mitochondria in cancer cells, which often have a more negative membrane potential.
A higher positive charge may lead to greater selectivity for cancer cells over normal cells, improving the therapeutic index.
Peptide Length: The length of anticancer peptides can significantly impact their efficacy.
- Shorter peptides (typically 10-30 amino acids) often demonstrate better penetration into tumor tissues and cancer cells.
- They may have reduced immunogenicity compared to longer peptides.
- Shorter lengths can facilitate easier synthesis and modification.

However, the optimal length can vary depending on the specific cancer target and mechanism of action. Some longer peptides might offer more complex interactions with cellular targets.

Boman Index: The Boman index, indicating the potential for protein-protein interactions, is relevant for anti-cancer activities:

- A higher Boman index suggests greater potential for intracellular interactions in cancer cells.
- This could lead to interference with key cellular processes specific to cancer cells, such as signal transduction or gene expression.
- Peptides with a higher Boman index might exhibit multiple mechanisms of action against cancer cells, potentially reducing the likelihood of resistance development.

On the other hand, a Boman index value of 1 or below suggests that the protein possesses a reduced risk of causing side effects and toxicity. The Boman index, with values less than one, can indicate relatively favorable interactions with cancer cells and, at the same time, exhibit lower toxicity by having weaker interactions with normal cells (38).

Hydrophobicity: The hydrophobicity of peptides is critical for their anti-cancer effects.

- Moderate hydrophobicity is often optimal, allowing for:
• Efficient interaction with and penetration of cancer cell membranes.
• Better discrimination between normal and cancer cell membranes.
- Highly hydrophobic peptides might show increased cytotoxicity but could lose selectivity for cancer cells.
- The right balance of hydrophobicity can enhance cellular uptake and intracellular distribution in cancer cells.

The prevalence of helical structures in the data suggests that this conformation is particularly important for anti-cancer activities. Helical structures can:

- Facilitate interaction with and disruption of cancer cell membranes.
- Potentially form pores in cancer cell membranes, leading to cell death.
- Enable better penetration into solid tumors.

In conclusion, the anti-cancer properties of these peptides are intricately linked to their structural characteristics. The optimal combination of positive net charge, appropriate length, balanced Boman index, and moderate hydrophobicity can lead to peptides with enhanced selectivity and efficacy against cancer cells. Future research focusing on fine-tuning these parameters could lead to the development of more potent and selective anti-cancer peptides, potentially offering new avenues for cancer therapy with reduced side effects compared to traditional chemotherapeutics.

The comprehensive analysis of these antimicrobial peptides reveals that their various characteristics - including length, charge, structure, Boman index, and hydrophobicity - are all well-suited for their anti-cancer activities. The peptide lengths observed are generally optimal for penetrating tumor tissues and cancer cells, while maintaining selectivity. Their net charges, predominantly positive, facilitate strong interactions with cancer cell membranes. The prevalence of helical structures enhances their ability to disrupt cancer cell membranes effectively. The Boman indices of these peptides suggest a good balance between membrane interaction and potential intracellular activities, which is crucial for diverse anti-cancer mechanisms. Additionally, their hydrophobicity levels appear to be in a range that allows for effective membrane penetration without excessive toxicity to normal cells. Collectively, these characteristics contribute to the peptides’ ability to target and eliminate cancer cells through various mechanisms, including membrane disruption, intracellular targeting, and potential signaling pathway interference. The combination of these properties makes these antimicrobial peptides particularly promising candidates for anti-cancer therapies, offering a multi-faceted approach to combating cancer cells while potentially minimizing harm to normal tissues (37, 39–41).

## 5 Mechanisms of action of AMPs in cancer

AMPs exert their anti-cancer effects through a multifaceted approach, targeting both cancer cells directly and the tumor microenvironment.

### 5.1 Direct cytotoxicity

AMPs exhibit direct cytotoxicity towards cancer cells through several mechanisms, primarily involving interactions with the cell membrane and subsequent induction of apoptosis. The membrane-disrupting activity of AMPs is a key factor in their anticancer effects. AMPs can bind to and insert into the lipid bilayer of cancer cell membranes, leading to membrane destabilization and eventual rupture. This disruption results in the leakage of intracellular contents and cell death. The cationic nature of many AMPs allows them to interact preferentially with the negatively charged phospholipids found in higher concentrations on the surface of cancer cells, enhancing their selectivity and efficacy. In addition to membrane disruption, AMPs can induce apoptosis in cancer cells. This process involves the activation of caspase pathways and the release of pro-apoptotic factors. Some AMPs can trigger the formation of membrane pores, leading to the influx of calcium ions and activation of calpain proteases, which in turn activate caspases and initiate apoptosis. Other AMPs may directly interact with intracellular targets, such as mitochondria, leading to the release of cytochrome c and the activation of the intrinsic apoptotic pathway. The specific structural features of AMPs play a crucial role in their cytotoxic activity. For instance, the amphipathic nature of many AMPs, characterized by a combination of hydrophobic and hydrophilic regions, allows them to interact effectively with the lipid bilayer. The length, charge distribution, and secondary structure of AMPs also influence their ability to disrupt membranes and induce apoptosis. Alpha-helical AMPs, for example, are particularly effective at penetrating and disrupting membranes due to their ability to adopt a helical conformation in the lipid environment (42–45).

### 5.2 Modulation of immune response

AMPs can stimulate the immune system to recognize and eliminate cancer cells, making them valuable agents in cancer immunotherapy. By modulating the immune response, AMPs can enhance the body’s natural defenses against cancer. One mechanism involves the activation of pattern recognition receptors (PRRs) on immune cells, such as Toll-like receptors (TLRs) and NOD-like receptors (NLRs). These receptors recognize AMPs and trigger signaling pathways that lead to the production of pro-inflammatory cytokines and the activation of immune cells. AMPs can also modulate the function of various immune cells, including dendritic cells, macrophages, and T cells. For example, AMPs can enhance the maturation and antigen-presenting capabilities of dendritic cells, leading to increased activation of T cells and improved immune surveillance of cancer cells. Additionally, AMPs can stimulate the production of cytokines such as interferon-gamma (IFN-γ) and tumor necrosis factor-alpha (TNF-α), which have potent antitumor effects. The ability of AMPs to modulate immune cell function and cytokine production makes them valuable tools in enhancing the immune response against cancer. By promoting a more robust and effective immune reaction, AMPs can help eliminate cancer cells and prevent tumor recurrence (32, 46, 47).

### 5.3 Targeting the tumor microenvironment

The tumor microenvironment plays a critical role in cancer progression, including processes such as angiogenesis, metastasis, and interactions with stromal cells. AMPs can impact the tumor microenvironment in several ways, disrupting these processes and enhancing therapeutic efficacy. One significant effect of AMPs on the tumor microenvironment is their ability to inhibit angiogenesis, the formation of new blood vessels that supply nutrients and oxygen to the tumor. AMPs can target endothelial cells lining blood vessels, disrupting their function and reducing vascular supply to the tumor. This vascular disruption can lead to tumor hypoxia and necrosis, impairing tumor growth and survival. AMPs can also influence metastasis, the spread of cancer cells to distant organs. By disrupting the interactions between cancer cells and the extracellular matrix, AMPs can inhibit the migration and invasion of cancer cells. Additionally, AMPs can target cancer-associated fibroblasts and other stromal cells, reducing their supportive functions and limiting tumor growth. Furthermore, AMPs can modulate the immune cells within the tumor microenvironment, such as regulatory T cells (Tregs) and myeloid-derived suppressor cells (MDSCs), which often suppress the immune response and promote tumor growth. By reducing the activity of these suppressive cells, AMPs can enhance the antitumor immune response and improve the efficacy of other therapeutic modalities (42, 44, 48).

In summary, the mechanisms of action of AMPs in cancer involve direct cytotoxicity, modulation of the immune response, and targeting of the tumor microenvironment. These multifaceted effects make AMPs promising agents in the development of novel anticancer therapies, offering potential for more effective and targeted treatment strategies.

## 6 *In vitro* assays for anticancer peptides

The efficacy of AMPs as anticancer agents has been extensively evaluated *in vitro*, providing foundational evidence for their potential in cancer therapy (49). In **Supplementary Table 1**, we summarized all reported AMPs with anticancer activity. Most of these reported peptides have been evaluated in *in vitro*. These *in vitro* assays have demonstrated the ability of AMPs to inhibit cancer cell proliferation, induce apoptosis, and disrupt cellular membranes across a variety of cancer cell lines. Here, we summarize key findings from *in vitro* studies involving several notable AMPs from prepared list. *In vitro* studies have shown that cathelicidin family, including LL-37, can effectively reduce the viability of breast cancer and ovarian cancer cells by inducing apoptosis and inhibiting cell migration. This peptide family disrupt the cancer cell membrane, leading to cell death, and has been observed to modulate the expression of genes involved in cancer progression (50). Magainins, as class of AMPs found in the amphibians, have been tested on colon cancer and gastric cancer cell lines, where it has demonstrated significant cytotoxicity. The mechanism involves pore formation in the cancer cell membrane, leading to increased permeability and subsequent cell lysis (51, 52). *In vitro* experiments with lactoferricin, an amphipathic, cationic peptide with dual biological activity (anti-microbial and anti-cancer), have shown inhibition of melanoma and leukemia cell lines. The peptide induces apoptosis through mitochondrial pathways and has been observed to inhibit angiogenesis by affecting endothelial cells (53). Studies have reported that Protegrin PG-1, a cysteine-rich peptide, s effective against prostate cancer and other carcinoma cell lines. It disrupts the membrane integrity of cancer cells, leading to rapid cell death, and has shown synergistic effects when used in combination with conventional chemotherapeutics (54). Dermaseptins, especially Dermaseptins -B2, has shown significant anticancer activity against a variety of cancer cell lines, including melanoma and breast cancer cells. *In vitro* studies reveal that this peptide exerts its effects primarily through membrane disruption and the induction of apoptosis, effectively reducing cancer cell viability and proliferation (15, 55–57). Cecropins family have been tested on bladder cancer and breast cancer cell lines, showing significant reduction in cell viability. The mechanism primarily involves disruption of the cancer cell membrane and induction of apoptosis (37, 58). *In vitro* studies with melittin have demonstrated its efficacy against lung and liver cancer cell lines. It acts by disrupting cellular membranes and inducing apoptotic pathways, and it has been utilized in nanoparticle delivery systems to enhance its selectivity for cancer cells (59). The aurein peptides, derived from frog skin, have demonstrated notable cytotoxic effects against cancer cell lines, such as prostate and breast cancer cells. These peptides function by disrupting cancer cell membranes and initiating apoptotic pathways, leading to cell death (16). Alloferons, originally isolated from insects, have been investigated for their anticancer properties. *In vitro* assays have shown that these peptides can induce apoptosis in leukemia and gastric cancer cells. They activate immune responses and enhance the cytotoxicity of immune cells against cancer cells (60). Synthetic hybrid peptide CE-MA has been shown to possess potent anticancer activity against a range of cancer cell lines, including lung and bladder cancers. It acts by disrupting cell membranes and enhancing apoptosis, demonstrating greater efficacy compared to its parent peptides alone (61). Maximin peptides, isolated from amphibians, have been studied for their effects on cancer cells such as melanoma and gastric cancer. *In vitro*, they disrupt cancer cell membranes and induce cell death, showing promise as potential anticancer agents (62). Temporin A and Temporin L, isolated from frog skin, have shown *in vitro* efficacy against breast and prostate cancer cell lines. They exert their anticancer effects through membrane disruption and the induction of apoptotic pathways, highlighting their potential in cancer therapy (63). Known for its broad-spectrum antimicrobial activity, indolicidin has also shown *in vitro* efficacy against leukemia and breast cancer cells. It induces apoptosis by triggering mitochondrial dysfunction and inhibiting cell cycle progression. Human Neutrophil Peptides (HNP-1, HNP-2, HNP-3) have demonstrated potent anticancer effects *in vitro* against lung and colorectal cancer cells. They act by disrupting cellular membranes and modulating inflammatory responses, which contribute to their anticancer activity (64). Synthetic, lysine-rich mutant of CRAMP-18 has been engineered to enhance its anticancer properties. *In vitro* studies have demonstrated its ability to disrupt cancer cell membranes and induce apoptosis in various cancer cell lines, including breast and colon cancer. The lysine-rich composition enhances its interaction with negatively charged cancer cell membranes, leading to increased cytotoxicity (65). Brevinins and heir derivatives have shown potent anticancer activity *in vitro*. They has demonstrated efficacy against melanoma and breast cancer cell lines by inducing membrane disruption and apoptosis. The presence of disulfide bonds (1S=S) contributes to its structural stability and anticancer activity. Synthetic mutant of Brevinin-1-AW, with a Q15K substitution (B1AW-K), exhibits enhanced anticancer activity compared to its parent peptide. *In vitro* assays have shown increased cytotoxic effects on prostate and pancreatic cancer cells, attributed to improved membrane interaction and apoptotic induction (66–68). A leucine-rich natural AMP from amphibians, Figainin 2BN has been effective in reducing viability in colon and lung cancer cell lines. Its mechanism involves membrane permeabilization and induction of apoptosis, highlighting its potential as a therapeutic agent (69). Picturin 1BN and Picturin 2BN, natural AMPs from amphibians, have shown promising *in vitro* anticancer activity against leukemia and glioblastoma cells. They function by disrupting cellular membranes and triggering apoptotic pathways, leading to significant reductions in cancer cell proliferation (70). Dermaseptins, derived from amphibians, have exhibited strong anticancer effects *in vitro*. They target a wide range of cancer cell lines, including skin and cervical cancers, by causing membrane disruption and subsequent apoptosis (71, 72). MPC-A5K, an analog of Mastoparan C, is lysine- and leucine-rich and has shown enhanced *in vitro* anticancer activity against gastric and prostate cancer cells. It achieves its effects through improved membrane interaction and increased induction of apoptotic pathways (73). Ranatuerins have been designed to improve anticancer activity. *In vitro* studies indicate their efficacy in inducing apoptosis and reducing proliferation in breast and liver cancer cells through enhanced membrane disruption (74). Raniseptin PL from amphibians has shown *in vitro* effectiveness against ovarian and lung cancer cell lines. It operates by disrupting cancer cell membranes and activating apoptotic mechanisms, leading to reduced tumor cell survival (75). Figainin 2PL and Hylin PL, derived from amphibian sources, demonstrate strong *in vitro* anticancer activity against a variety of cancer cell lines, including leukemia and colon cancer. Their mode of action involves membrane permeabilization and apoptosis induction (75). StigA6 and StigA16, synthetic analogs of Stigmurin, have been optimized for enhanced anticancer activity *in vitro*. They exhibit potent effects against colorectal and pancreatic cancer cells by disrupting membranes and inducing apoptosis (41). AP-64, identified as C5orf46, has shown promise *in vitro* against hematological and solid tumors. It exerts its effects through membrane disruption and the activation of apoptotic pathways, making it a candidate for further anticancer research (40). AaeAP1a and AaeAP2a, lysine-rich synthetic analogs, have been developed to enhance anticancer activity. *In vitro* studies show their effectiveness in reducing viability in breast and colon cancer cells by disrupting membranes and inducing apoptosis (39). The *in vitro* studies of these AMPs provide compelling evidence of their direct cytotoxic effects on cancer cells and support their potential as novel anticancer agents. These findings lay the groundwork for further exploration in preclinical animal models and clinical trials, with the aim of optimizing their use in cancer therapy.

## 7 Preclinical studies and clinical trials

The therapeutic potential of AMPs in cancer treatment has been extensively explored in preclinical studies, with promising results paving the way for clinical trials.

### 7.1 Preclinical studies in animal models

Preclinical studies in animal models have provided substantial evidence supporting the efficacy of AMPs against various types of cancer. These studies have investigated the effects of AMPs on a wide range of cancers, including solid tumors and hematologic malignancies, and have explored different treatment regimens and delivery strategies.

Solid Tumors: AMPs have demonstrated significant antitumor activity against various solid tumors in preclinical models. LL-37 has shown efficacy against breast cancer in mice, reducing tumor growth and metastasis (76). Another study investigated the effects of magainin II on colon cancer in a rat model, reporting significant tumor regression and improved survival (77). Lactoferricin B has also exhibited potent antitumor activity against melanoma in mice, inhibiting tumor growth and angiogenesis (78). Furthermore, Protegrin PG-1 has demonstrated efficacy against prostate cancer in a mouse model, reducing tumor volume and improving survival (54).

Hematologic Malignancies: AMPs have also shown promise in the treatment of hematologic malignancies in preclinical studies. The LTX-315 has demonstrated potent antileukemic activity *in vitro* and *in vivo*, inducing apoptosis in leukemia cells and reducing tumor burden in mouse models (79). Another study investigated the effects of PFR-1 on lymphoma in mice, reporting significant tumor regression and improved survival (80). Additionally, Cyclic AMP has shown efficacy against multiple myeloma in a mouse model, reducing tumor growth and enhancing the effects of conventional chemotherapy (81).

Treatment Regimens and Outcomes: Preclinical studies have explored various treatment regimens and delivery strategies for AMPs in cancer therapy. Single-agent therapy with AMPs has demonstrated significant antitumor activity in several studies (82–85). However, combination therapy approaches have also shown promise. Targeted delivery strategies have also been explored to enhance the efficacy and specificity of AMPs in cancer treatment (86, 87). One study investigated the use of a tumor-targeting peptide conjugated to the AMP cecropin A, demonstrating improved tumor localization and antitumor activity in a mouse model of breast cancer (86). Another study utilized a nanoparticle delivery system to target the AMP melittin to lung cancer cells, resulting in enhanced tumor regression and reduced systemic toxicity in mice (55).

The therapeutic outcomes observed in preclinical studies have been promising, with AMPs demonstrating significant antitumor effects across various cancer types. Tumor regression, metastasis inhibition, and improved survival have been consistently reported in animal models treated with AMPs (76, 77, 81). These findings highlight the potential of AMPs as novel therapeutic agents for cancer treatment and provide a strong foundation for further clinical development.

In conclusion, preclinical studies in animal models have provided compelling evidence supporting the efficacy of AMPs in cancer treatment. These studies have demonstrated the antitumor activity of AMPs against a wide range of solid tumors and hematologic malignancies, explored various treatment regimens and delivery strategies, and reported promising therapeutic outcomes. However, further research is needed to optimize AMP-based therapies, elucidate their mechanisms of action, and address potential challenges in translating these findings to human clinical trials.

### 7.2 Clinical trials in humans

The promising preclinical results have led to the initiation of clinical trials in humans to evaluate the safety and efficacy of AMPs for cancer therapy. Several clinical trials are currently ongoing, investigating the use of AMPs for various cancer types. These trials span different phases, each with specific objectives and patient populations (82, 88, 89). Clinical trials are targeting a range of patient populations, including those with advanced cancers who have exhausted conventional therapies, such as in the LTX-315 trial focusing on patients with metastatic solid tumors. AMPs are also being explored as potential treatments for patients with drug-resistant cancers. Additionally, some trials are exploring the use of AMPs as adjuvant therapies in early-stage cancers, such as the bovine lactoferrin trial in head and neck cancer patients aiming to prevent recurrence in those who have undergone primary treatment Preliminary results from early-phase clinical trials are encouraging. In the Phase I trial of LTX-315, 50% of evaluable patients showed stable disease, and one patient metastatic soft tissue sarcoma experienced a partial response (90). These results suggest that AMPs may have significant therapeutic potential, particularly when combined with other immunotherapies. However, it’s important to note that larger-scale, late-stage clinical trials are needed to confirm these findings and establish the true efficacy of AMPs in cancer treatment. The field also faces challenges in optimizing delivery methods, reducing potential systemic toxicity, and overcoming regulatory hurdles In conclusion, while the clinical development of AMPs for cancer therapy is still in its early stages, the ongoing trials and preliminary results offer promising avenues for future research and potential new treatment options for cancer patients. The diverse range of AMPs being investigated, from naturally derived peptides like LL-37 to synthetic variants like LTX-315, highlights the versatility of this approach. As research progresses, it will be crucial to address challenges such as peptide stability, targeted delivery, and potential immunogenicity to fully realize the therapeutic potential of AMPs in cancer treatment.

### 7.3 Challenges and future directions

The development of antimicrobial peptides (AMPs) for clinical application in cancer therapy faces several challenges that must be addressed to translate these promising agents into effective treatments. Key issues include stability, delivery, and potential toxicity, each of which poses significant hurdles for the successful clinical use of AMPs.

#### 7.3.1 Challenges in AMP development

Stability: One of the primary challenges in AMP development is ensuring their stability in biological environments. AMPs can be susceptible to degradation by enzymes such as proteases, which can limit their efficacy and duration of action. Additionally, the chemical stability of AMPs in the presence of physiological conditions, such as changes in pH and temperature, must be considered to maintain their structural integrity and functional activity.

Delivery: Effective delivery of AMPs to the target site within the body is another major challenge. Systemic administration of AMPs can lead to off-target effects and toxicity due to their interaction with healthy tissues. Localized delivery methods, such as direct injection into tumors, can improve specificity but may not be feasible for disseminated cancers. Novel delivery systems, including nanoparticles, liposomes, and polymeric carriers, are being explored to enhance the targeting and efficacy of AMPs while reducing systemic exposure.

Potential Toxicity: While AMPs have shown selectivity for cancer cells, concerns remain about their potential toxicity to healthy tissues. Some AMPs can induce inflammation and immune reactions, particularly at higher doses, which can lead to adverse effects. Understanding the mechanisms of AMP-induced toxicity and developing strategies to minimize these effects are crucial for their safe and effective use in cancer therapy (21, 86, 91).

#### 7.3.2 Future directions for research and development

To overcome these challenges and translate AMPs into effective cancer therapies, several future directions for research and development are being pursued.

Structural Modifications: One approach is to modify the structure of AMPs to enhance their stability and reduce toxicity. This can involve altering the peptide sequence, introducing chemical modifications, or engineering fusion proteins that combine the anticancer activity of AMPs with other functional domains. These modifications can improve the resistance of AMPs to proteolytic degradation and enhance their selectivity for cancer cells.

Delivery Systems: Developing advanced delivery systems is another key area of research. Nanotechnology-based approaches, such as encapsulating AMPs within nanoparticles or conjugating them to targeting ligands, can improve their delivery to tumors and reduce systemic exposure. These delivery systems can also be designed to release AMPs in a controlled manner, enhancing their therapeutic window and minimizing side effects.

Combination Therapies: Exploring the use of AMPs in combination with other therapeutic modalities is a promising strategy to enhance their efficacy. Combining AMPs with traditional chemotherapy, radiotherapy, or immunotherapy can exploit synergistic effects and improve treatment outcomes. This approach can also help overcome resistance mechanisms and target multiple pathways involved in cancer progression.

Clinical Trials and Regulatory Approval: Advancing AMPs through clinical trials and obtaining regulatory approval are critical steps for their translation into clinical practice. Future clinical trials should focus on optimizing dosing regimens, assessing long-term safety and efficacy, and demonstrating the clinical benefit of AMPs in specific cancer types. Collaborations between academia, industry, and regulatory agencies will be essential to navigate the complex process of drug development and approval (21, 32, 92).

In summary, while the development of AMPs for clinical application in cancer therapy faces significant challenges, future research and development efforts are focused on overcoming these hurdles. By addressing issues related to stability, delivery, and potential toxicity, and by exploring novel strategies such as structural modifications, advanced delivery systems, and combination therapies, AMPs have the potential to become effective and safe anticancer agents.

## 8 Applications of AMPs in cancer therapy

### 8.1 Single-agent therapy

Single-Agent Therapy involves the use of a single therapeutic agent, in this case, antimicrobial peptides (AMPs), as standalone agents for cancer treatment. This approach leverages the unique properties of AMPs, such as their direct cytotoxicity and ability to modulate the immune response, to target and eliminate cancer cells.

The potential of different AMP classes for specific cancer types is an area of active research. For instance, certain cationic AMPs, known for their membrane-disrupting activity, have shown efficacy in treating solid tumors with high levels of negatively charged phospholipids on their cell surfaces. Examples include α-helical peptides like melittin and magainin, which have been investigated for their anticancer effects in breast, prostate, and colon cancers.

Other AMP classes, such as defensins and cathelicidins, exhibit additional mechanisms of action beyond membrane disruption. These peptides can induce apoptosis, modulate the immune response, and interact with intracellular targets, making them suitable for a broader range of cancer types. For example, α-defensins have been studied for their potential in treating hematological malignancies, while LL-37, a cathelicidin peptide, has shown promise in melanoma and glioblastoma.

The use of AMPs as Single-Agent Therapy offers several advantages, including their broad-spectrum activity, potential for reduced resistance, and ability to target multiple pathways within cancer cells. However, challenges such as stability, delivery, and potential toxicity must be addressed to optimize their efficacy and safety as standalone agents (93–96).

### 8.2 Combination therapy

Combination therapy involves the use of AMPs in conjunction with other cancer therapies, such as chemotherapy, radiotherapy, and immunotherapy. This approach aims to exploit synergistic effects and enhance the overall efficacy of treatment regimens.

The rationale for combining AMPs with other therapies is based on their complementary mechanisms of action. For example, AMPs can enhance the cytotoxic effects of chemotherapy by disrupting tumor cell membranes and facilitating the entry of chemotherapeutic agents into cancer cells. Similarly, AMPs can potentiate the effects of radiotherapy by inducing apoptosis and modulating the tumor microenvironment, thereby improving the response to radiation.

In the context of immunotherapy, AMPs can stimulate the immune system to recognize and eliminate cancer cells. By activating pattern recognition receptors and modulating immune cell function, AMPs can enhance the efficacy of checkpoint inhibitors and other immunotherapeutic agents. Preclinical studies have demonstrated synergistic effects when AMPs are combined with immunotherapy, leading to improved tumor regression and survival rates.

Clinical evidence supporting the benefits of AMP combination therapies is emerging. Early-phase clinical trials have shown promising results, with some patients experiencing enhanced tumor responses and prolonged survival when AMPs are used in combination with conventional therapies. These findings highlight the potential of AMP combination therapies to improve treatment outcomes and overcome resistance mechanisms in cancer (97–100).

### 8.3 Targeted drug delivery

Targeted drug delivery involves the use of AMPs as delivery vehicles to specifically target cancer cells and enhance the efficiency of drug delivery. This approach leverages the natural properties of AMPs, such as their ability to bind to and penetrate cancer cell membranes, to deliver therapeutic payloads directly to tumor cells.

AMPs can be engineered to carry cytotoxic agents, imaging contrast agents, or therapeutic genes, allowing for targeted and controlled release within cancer cells. For example, AMPs can be conjugated with chemotherapeutic drugs, such as doxorubicin or paclitaxel, to create peptide-drug conjugates that selectively deliver the drugs to tumor cells. These conjugates can reduce systemic exposure to the drugs, minimizing side effects and improving the therapeutic index.

Additionally, AMPs can be engineered to target specific receptors or surface markers overexpressed on cancer cells. By incorporating targeting ligands, such as monoclonal antibodies or small molecules, into the AMP structure, the peptide can be directed to bind preferentially to cancer cells, enhancing its specificity and efficacy. This targeted approach can also reduce off-target effects and improve the overall safety profile of the therapy.

In summary, the applications of AMPs in cancer therapy encompass Single-Agent Therapy, combination therapy, and targeted drug delivery. Each approach leverages the unique properties of AMPs to enhance their anticancer effects and improve treatment outcomes. By addressing challenges related to stability, delivery, and potential toxicity, and by exploring innovative strategies such as combination therapies and targeted drug delivery, AMPs have the potential to become valuable agents in the fight against cancer (101–105).

## 9 Conclusion and future perspectives

### 9.1 Summary of key findings

This review has highlighted the promising potential of antimicrobial peptides (AMPs) as cancer therapeutics. Key findings from preclinical studies and early-phase clinical trials indicate that AMPs exhibit direct cytotoxicity, modulate the immune response, and can target the tumor microenvironment, making them valuable agents in the fight against cancer. The diverse mechanisms of action of AMPs, coupled with their broad-spectrum activity and potential for reduced resistance, position them as a novel class of anticancer therapeutics with significant advantages over conventional treatments.

### 9.2 Future directions and research needs

Several critical areas for future research in AMP development have been identified to fully realize their potential as effective cancer therapies. These include:

Optimization of AMP Properties: Further research is needed to optimize the structural and functional properties of AMPs to enhance their stability, selectivity, and efficacy. This can involve structural modifications, such as altering peptide sequences, introducing chemical modifications, or engineering fusion proteins, to improve their resistance to proteolytic degradation and reduce potential toxicity.

Development of Novel Delivery Strategies: The development of advanced delivery systems is crucial for improving the targeting and efficacy of AMPs while minimizing systemic exposure. Novel delivery strategies, such as nanotechnology-based approaches, liposomes, and polymeric carriers, can enhance the delivery of AMPs to tumors and reduce off-target effects. Additionally, targeted delivery systems that incorporate specific ligands to preferentially bind to cancer cells can improve the specificity and efficacy of AMP therapy.

Exploration of Synergistic Combination Therapies: The use of AMPs in combination with other therapeutic modalities, such as chemotherapy, radiotherapy, and immunotherapy, offers a promising strategy to enhance their efficacy. Future research should focus on identifying optimal combination regimens and exploring the synergistic effects of these therapies. Preclinical and clinical studies should be conducted to assess the safety, efficacy, and clinical benefit of AMP combination therapies.

### 9.3 Conclusion

In conclusion, the significant potential of AMPs to revolutionize cancer treatment and improve patient outcomes cannot be overstated. With ongoing research and development efforts focused on optimizing AMP properties, developing novel delivery strategies, and exploring synergistic combination therapies, AMPs have the potential to become valuable agents in the fight against cancer. By addressing the challenges associated with AMP development and leveraging their unique properties, we can advance the field of cancer therapy and provide new hope for patients in need of effective and targeted treatments.
