# Supplementary material for: From defense to offense: antimicrobial peptides as promising therapeutics for cancer
Source: Front Oncol. 2024 Oct 9;14:1463088. doi: 10.3389/fonc.2024.1463088 (PMC11496142; doi:10.3389/fonc.2024.1463088)
Supplement: Supplementary file 1 [file Table1.docx]

Table 1. Summary of Antimicrobial Peptides (AMPs) with Anti-Cancer Activity

| **Name** | **Sequence** | **Source** | **Length** | **Net Charge** | **Hydrophobic residue%** | **Boman index** | **3D Structure** | **Reference** |
| --- | --- | --- | --- | --- | --- | --- | --- | --- |
| Dermaseptin-B2 (XXA, DRS-B2, Dermaseptin B2, DRS B2, DS bII, ADENOREGULIN; natural AMPs; Ala-rich; UCLL1c; frog, amphibians, animals) | GLWSKIKEVGKEAAKAAAKAAGKAALGAVSEAV | skin, [Giant leaf frog](https://amphibiaweb.org/cgi/amphib_query?where-genus=Phyllomedusa&where-species=bicolor), Phyllomedusa bicolor, South America | 33 | 4 | 54% | 0.23 | Helix | (1) |
| Aurein 1.2 (natural AMPs; XXA, UCLL1c; frog, amphibians, animals; BBMm; ZZH) | GLFDIIKKIAESF | Southern bell frog Litoria aurea and Litoria raniformis, Australia | 13 | 1 | 53% | 0.12 | Helix | (2) |
| Aurein 2.1 (natural AMPs; XXA, UCLL1c; frog, amphibians, animals) | GLLDIVKKVVGAFGSL | Southern bell frog Litoria aurea or Litoria raniformis, Australia | 16 | 2 | 56% | -1.01 | Unknown | (2) |
| Aurein 2.2 (natural AMPs; XXA, UCLL1c; frog, amphibians, animals; BBMm) | GLFDIVKKVVGALGSL | Southern bell frog Litoria aurea or Litoria raniformis, Australia | 16 | 2 | 56% | -1.01 | Helix | (2) |
| Aurein 2.3 (natural AMPs; XXA, UCLL1c; frog, amphibians, animals) | GLFDIVKKVVGAIGSL | Southern bell frog Litoria aurea or Litoria raniformis, Australia | 16 | 2 | 56% | -1.01 | Helix | (2) |
| Aurein 2.4 (natural AMPs; XXA, UCLL1c; frog, amphibians, animals) | GLFDIVKKVVGTLAGL | Southern bell frog Litoria aurea or Litoria raniformis, Australia | 16 | 2 | 56% | -1.06 | Unknown | (2) |
| Aurein 2.5 (natural AMPs; XXA, UCLL1c; frog, amphibians, animals, BBMm) | GLFDIVKKVVGAFGSL | Southern bell frog Litoria aurea or Litoria raniformis, Australia | 16 | 2 | 56% | -0.89 | Helix | (2) |
| Aurein 2.6 (natural AMPs; XXA, UCLL1c; frog, amphibians, animals) | GLFDIAKKVIGVIGSL | Southern bell frog Litoria aurea or Litoria raniformis, Australia | 16 | 2 | 56% | -1.06 | Unknown | (2) |
| Aurein 3.1 (natural AMPs; XXA, UCLL1c; frog, amphibians, animals) | GLFDIVKKIAGHIAGSI | Southern bell frog Litoria aurea or Litoria raniformis, Australia | 17 | 2 | 52% | -0.59 | Unknown | (2) |
| Aurein 3.2 (natural AMPs; XXA, UCLL1c; frog, amphibians, animals) | GLFDIVKKIAGHIASSI | Southern bell frog Litoria aurea or Litoria raniformis, Australia | 17 | 2 | 52% | -0.34 | Unknown | (2) |
| Aurein 3.3 (natural AMPs; XXA, UCLL1c; frog, amphibians, animals) | GLFDIVKKIAGHIVSSI | Southern bell frog Litoria aurea or Litoria raniformis, Australia | 17 | 2 | 52% | -0.47 | Unknown | (2) |
| Antiviral protein Y3 (natural AMPs; edible fungi, fungii; UCSS1a) | AACARFIDDFCDTLTPNIYRPRDNGQRCYAVNGHRCDFTVFNTNNGGNPIRASTPNCKTVLRTAANRCPTGGRGKINPNAPFLFAIDPNDGDCSTNF | Golden oyster mushroom, Pleurotus citrinopileatus | 97 | 4 | 35% | 2.44 | Unknown | (3) |
| Alloferon 2 (Alloferon-2; Gly-rich, classic His-rich; natural non-AMPs, insects, arthropods, invertebrates, animals; BBII) | GVSGHGQHGVHG | Blow fly Calliphora vicina | 12 | 3 | 16% | 0.84 | Unknown | (4) |
| Alloferon 1 (Alloferon-1; natural AMPs; metallo-AMPs; Gly-rich, His-rich, insects, arthropods, invertebrates, animals; BBII, Derivatives: ZL-2) | HGVSGHGQHGVHG | Blow fly Calliphora vicina | 13 | 4 | 15% | 1.13 | Rich | (4) |
| Lactoferricin B (LfcinB, natural AMPs; UCSS1a; 1S=S, cattle, ruminant, animals, ZZHa; BBL; Derivatives: lactoferrin peptide 2; LTX-302; LfcinB6; MPLfcinB6; JJsn) | FKCRRWQWRMKKLGAPSITCVRRAF | cattle, Bos taurus | 25 | 8 | 48% | 2.75 | beta | (5) |
| Cecropin A(1-8)-Magainin 2(4-12) hybrid peptide (CE-MA) (Lys-rich; CAMEL; synthetic20, UCLL; BBL) | KWKLFKKIKFLHSAKKF | hybrid peptide | 17 | 8 | 47% | 1.12 | Helix | (6) |
| Plantaricin A (PlnA, natural AMPs; bacteriocin/pheromone, Gram-positive bacteria, prokaryotes; Other truncated forms: PlnA 23-mer; PlnA 22-mer) | KSSAYSLQMGATAIKQVKKLFKKWGW | Lactiplantibacillus plantarum; (old) Lactobacillus plantarum C11, WCFS1, V90 | 26 | 6 | 42% | 0.81 | Helix | (7) |
| Bombinin-like peptide 7 (BLP-7, natural AMPs; UCLL1a; toad, amphibians, animals) | GIGGALLSAGKSALKGLAKGLAEHFAN | [Oriental fire-bellied toad/frog](http://www.frogforum.net/content/fire-bellied-toad-care-breeding-bombina-orientalis-relatives-123/), Bombina orientalis, Asia | 27 | 3 | 48% | -0.27 | Unknown | (8) |
| Maximin 1 (natural AMPs; UCLL1c; XXA; toad, amphibians, animals; ZZS) | GIGTKILGGVKTALKGALKELASTYAN | [Chinese red belly toad](http://amphibiaweb.org/cgi/amphib_query?where-genus=Bombina&where-species=maxima), Bombina maxima , Yunnan, China, Asia | 27 | 4 | 40% | 0.05 | Helix | (9) |
| Maximin 3 (natural AMPs; UCLL1a; toad, amphibians, animals; ZZHa; ZZS) | GIGGKILSGLKTALKGAAKELASTYLH | [Chinese red belly toad](http://amphibiaweb.org/cgi/amphib_query?where-genus=Bombina&where-species=maxima), Bombina maxima , Yunnan, China, Asia | 27 | 4 | 40% | -0.02 | Helix | (9) |
| Maximin 4 (UCLL1c; XXA; natural AMPs; Maximin-4; toad, amphibians, animals; ZZS) | GIGGVLLSAGKAALKGLAKVLAEKYAN | [Chinese red belly toad](http://amphibiaweb.org/cgi/amphib_query?where-genus=Bombina&where-species=maxima), Bombina maxima , Yunnan, China, Asia | 27 | 4 | 51% | -0.51 | Helix | (9) |
| Maximin 5 (natural AMPs; UCLL1a; toad, amphibians, animals) | SIGAKILGGVKTFFKGALKELASTYLQ | [Chinese red belly toad](http://amphibiaweb.org/cgi/amphib_query?where-genus=Bombina&where-species=maxima), Bombina maxima , Yunnan, China, Asia | 27 | 3 | 44% | -0.07 | Unknown | (9) |
| Brevinin-1EMb (Gaegurin-6, Gaegurin 6, GGN6, natural AMPs; frog, amphibians, animals; XXU; 1S=S, UCSS1a) | FLPLLAGLAANFLPTIICKISYKC | skin, Korean wrinkled frog Rana rugosa, Asia | 24 | 2 | 62% | -1.26 | Unknown | (10) |
| Temporin A (temporin-1Ta; temporin-Ta; TA; natural AMPs; Leu-rich; XXA; UCLL1c; frog, amphibians, animals; ZZP) | FLPLIGRVLSGIL | European common frog, Rana temporaria | 13 | 2 | 61% | -1.54 | Helix | (11) |
| Temporin L (Temporin-L, temporin-1Tl; temporin-Tl; TL; TemL, XXA, natural AMPs; UCLL1c; frog, amphibians, animals; BBL; JJsn; BBMm; eTL [5-9]: engineered temporin L) | FVQWFSKFLGRIL | European common frog, Rana temporaria | 13 | 3 | 61% | -0.12 | Helix | (11) |
| Cecropin B (natural AMPs; UCLL1a; insects, arthropods, invertebrates, animals) | KWKIFKKIEKVGRNIRNGIIKAGPAVAVLGEAKAL | Chinese oak silk moth, Antheraea pernyi | 35 | 7 | 48% | 0.88 | Unknown | (12) |
| Cecropin 2 (Md-Cec; Mdc; M. domestica cecropin; natural AMPs; UCLL1, insects, arthropods, invertebrates, animals; BBL; BBMm; BBN) | GWLKKIGKKIERVGQHTRDATIQTIGVAQQAANVAATLK | the Medfly, Ceratitis capitata; also housefly, Musca domestica | 39 | 5 | 41% | 1.49 | Helix | (13) |
| Cecropin A (XXA; natural AMPs; insects, arthropods, invertebrates, animals; ZZHa; ZZP; UCLL1c) | KWKLFKKIEKVGQNIRDGIIKAGPAVAVVGQATQIAK | jiant silk moth, Hyalophora cecropia | 37 | 7 | 45% | 0.84 | Helix | (14) |
| SK84 (natural AMPs; insects, arthropods, invertebrates, animals) | SQLGDLGSGAGQGGGGGGSIRAAGGAFGKLEAAREEEFFYKKQKEQLERLKNDQIHQAEFHHQQIKEHEEAIQRHKDFLNNLHK | fly, Drosophila virilis | 84 | 5 | 28% | 2.5 | Rich | (15) |
| Magainin 2 (Magainin II, PGS; Hebrew "shield"; natural AMPs; UCLL1a; frog, amphibians, animals. BBMm; BBL; Derivatives: MSI-99; st7-5; stapled peptides) | GIGKFLHSAKKFGKAFVGEIMNS | skin; Stomach, [African clawed frog](http://amphibiaweb.org/cgi/amphib_query?where-genus=Xenopus&where-species=laevis), Xenopus laevis, Africa | 23 | 3 | 43% | 0.41 | Helix | (16) |
| Melittin (XXA; natural AMPs; UCLL1c; insects, arthropods, invertebrates, animals; ZZHa, StrAR, BBL; ZZP; BBMm) | GIGAVLKVLTTGLPALISWIKRKRQQ | Honeybee venom, Apis mellifera (also A. florea, A. cerana) | 26 | 6 | 46% | 0.57 | Helix | (17) |
| Indolicidin (IR13; APB-13; XXA, natural AMPs; UCLL1c; Trp-rich, cathelicidin, cattle, ruminant, mammals; animals; BBN;BBPP/BBII; Derivatives: CP-11, MBI-549, Omiganan;MBI 226;MBI-226, clinical trials | ILPWKWPWWPWRR | bovine neutrophils, cattle, Bos taurus | 13 | 4 | 53% | 1.06 | nonhelixbeta | (18) |
| Tritrpticin (tritrypticin, SN13, peptide 1; natural AMPs; Trp-rich; UCLL1, porcine cathelicidin; mammals, animals; SeqAR; Derivatives) | VRRFPWWWPFLRR | pig | 13 | 4 | 53% | 2.9 | nonhelixbeta | (19) |
| Dermaseptin-B3 (DRS-B3; Dermaseptin B3, DRS B3, natural AMPs; UCLL1a; frog, amphibians, animals) | ALWKNMLKGIGKLAGQAALGAVKTLVGA | South American frog, Phyllomedusa bicolor | 28 | 4 | 57% | -0.74 | Unknown | (20) |
| Pleurocidin (Ple; NRC-4, NRC-04; WF2; XXA; natural AMPs; fish, animals, UCLL1a) | GWGSFFKKAAHVGKHVGKAALTHYL | the skin mucous secretions, Winter flounder, Pleuronectes americanus | 25 | 7 | 44% | 0.2 | Helix | (21) |
| human neutrophil peptide-1 (HNP-1, HNP1, natural AMPs; alpha Defensin, DEFA1B; UCSS1a; lectin; primates, mammals, animals; XXX; ZZHh, BBS; BBL; BBW; 3S=S) | ACYCRIPACIAGERRYGTCIYQGRLWAFCC | neutrophils; natural killer cells, monocytes; airway, saliva; Homo sapiens | 30 | 3 | 53% | 1.07 | Beta | (22) |
| human neutrophil peptide-2 (HNP-2, HNP2, natural AMPs; alpha Defensin, lectin; UCSS1a; primates, mammals, animals; ZZHh, BBS; 3S=S; BBMm) | CYCRIPACIAGERRYGTCIYQGRLWAFCC | neutrophils; natural killer cells, monocytes; sairway, aliva; Homo sapiens | 29 | 3 | 51% | 1.17 | Beta | (22) |
| human neutrophil peptide-3 (HNP-3, HNP3, natural AMPs; alpha Defensin, UCSS1a; primates, mammals, animals; lectin; ZZHh, BBS; 3S=S) | DCYCRIPACIAGERRYGTCIYQGRLWAFCC | neutrophils; natural killer cells, monocytes; airway, saliva; Homo sapiens | 30 | 2 | 50% | 1.42 | Beta | (22) |
| Gomesin (Gm, Arg-rich; natural AMPs; Spiders, arachnids, Chelicerata, arthropods, invertebrates, animals; XXA, ZZP, XXQ, 2S=S; UCSS1a) | QCRRLCYKQRCVTYCRGR | Hemocytes, Acanthoscurria gomesiana | 18 | 6 | 33% | 4.39 | Beta | (23) |
| Hepcidin (Hepcidin 20; natural AMPs; Cys-rich 40%; UCSS1a; human, primates, mammals, animals; 4S=S) | ICIFCCGCCHRSKCGMCCKT | Homo sapiens | 20 | 3 | 60% | 0.46 | Beta | (24) |
| Mastoparan B (Mastoparan-B, MP-B; MPB; XXA; natural AMPs; Lys-rich; insects, arthropods, invertebrates, animals; UCLL1c) | LKLKSIVSWAKKVL | Hornet, Vespa basalis | 14 | 5 | 57% | -0.2 | Helix | (25) |
| Mastoparan-L (Mastoparan L, MP L, natural AMPs; Ala-rich; Leu-rich; insects, arthropods, invertebrates, animals; XXA, UCLL1c) | INLKALAALAKKIL | Venom, Vespula lewisii | 14 | 4 | 71% | -0.96 | Helix | (26) |
| Nisin Z (natural AMPs; lantibiotic, type 1, class 1 bacteriocin, Gram-positive bacteria, prokaryotes; XXT5; XXW3; UCSS1b; Variants: Nisin ZP) | ITSISLCTPGCKTGALMGCNMKTATCNCSIHVSK | Lactococcus lactisNIZO 221 86 | 34 | 3 | 44% | 0.43 | Unknown | (27) |
| Nisin A (NisaplinTM, ChrisinTM, food additive E234; natural AMPs; lantibiotic, class 1 bacteriocin, Gram-positive bacteria, prokaryotes; XXO; XXT5; XXW3; UCSS1b; BBW; BBMm; JJsn; Variants: nisin AP) | ITSISLCTPGCKTGALMGCNMKTATCHCSIHVSK | Streptococcus lactis, reclassified as Lactococcus lactis | 34 | 5 | 44% | 0.37 | nonhelixbeta | (28) |
| Tachyplesin I (Tachyplesin 1, Tachyplesin-1; Tac; TP1; Arg-rich; natural AMPs; UCSS1a; Horseshoe Crab, arachnids, Chelicerata, arthropods, invertebrates, animals, XXA, ZZHa; BBMm; BBL; 2S=S) | KWCFRVCYRGICYRRCR | hemocytes, Southeast Asia, Tachypleus tridentatus; Tachypleus gigas; Carcinoscorpius rotundicauda | 17 | 7 | 47% | 3.53 | Beta | (29) |
| Pyrularia thionin (Pp-TH, Pp-defensin? natural AMPs; UCSS1a; 4S=S; flowering seed plants) | KSCCRNTWARNCYNVCRLPGTISREICAKKCDCKIISGTTCPSDYPK | nuts, Pyrularia pubera | 47 | 6 | 36% | 2.32 | Bridge | (30) |
| Caerin 1.1 (C 1.1.; Cae-1, XXA, natural AMPs; UCLL1c; frog, amphibians, animals; ZZHa; ZZP) | GLLSVLGSVAKHVLPHVVPVIAEHL | Australian green tree frog, Litoria splendida; Litoria rothii | 25 | 4 | 56% | -1.04 | Helix | (31) |
| Caerin 1.3 (XXA, natural AMPs; UCLL1c; frog, amphibians, animals) | GLLSVLGSVAQHVLPHVVPVIAEHL | Australian frog Litoria caerula | 25 | 3 | 56% | -1.04 | Unknown | (32) |
| Caerin 1.5 (XXA, Val-rich; natural AMPs; UCLL1c; frog, amphibians, animals) | GLLSVLGSVVKHVIPHVVPVIAEHL | Australian frog Litoria caerula | 25 | 4 | 56% | -1.13 | Unknown | (32) |
| Caerin 1.6 (XXA, natural AMPs; UCLL1c; frog, amphibians, animals; Caerin 1.6.1: inactive ) | GLFSVLGAVAKHVLPHVVPVIAEK | Orange-thighed frog, Litoria xanthomera, Australia | 24 | 4 | 58% | -0.98 | Unknown | (33) |
| Caerin 1.7 (XXA, natural AMPs; UCLL1c; frog, amphibians, animals) | GLFKVLGSVAKHLLPHVAPVIAEK | Orange-thighed frog, Litoria xanthomera, Australia | 24 | 5 | 54% | -0.61 | Unknown | (33) |
| Caerin 1.8 (XXA, natural AMPs; UCLL1c; frog, amphibians, animals; ZZP) | GLFKVLGSVAKHLLPHVVPVIAEK | [Blue-thighed frog](http://www.uniprot.org/taxonomy/86064), Litoria chloris, Australia | 24 | 5 | 54% | -0.71 | Unknown | (34) |
| Caerin 1.9 (XXA, natural AMPs; UCLL1c; frog, amphibians, animals; ZZHa) | GLFGVLGSIAKHVLPHVVPVIAEK | [Blue-thighed frog](http://www.uniprot.org/taxonomy/86064), Litoria chloris, Australia | 24 | 4 | 54% | -0.98 | Unknown | (34) |
| Maculatin 1.1 (Mac1; XXA, natural AMPs; UCLL1c; frog, amphibians, animals; ZZHa) | GLFVGVLAKVAAHVVPAIAEHF | skin secretions, Litoria genimaculate, Litoria eucnemis, Australia | 22 | 3 | 68% | -1.37 | Helix | (35) |
| Maculatin 1.2 (Ala-rich; XXA, natural AMPs; UCLL1c; frog, amphibians, animals) | GLFVGLAKVAAHNNPAIAEHFQA | Litoria genimaculate, Australia | 23 | 3 | 56% | -0.04 | Unknown | (35) |
| Maculatin 2.1 (XXA, natural AMPs; UCLL1c; frog, amphibians, animals) | GFVDFLKKVAGTIANVVT | Litoria genimaculate, Litoria eucnemis, Australia | 18 | 2 | 55% | -0.32 | Unknown | (35) |
| Maculatin 3.1 (XXA, natural AMPs; UCLL1c; frog, amphibians, animals) | GLLQTIKEKLESLESLAKGIVSGIQA | Litoria genimaculate, Australia | 26 | 1 | 42% | 0.42 | Unknown | (35) |
| CRAMP-1 (mouse cathelin-related antimicrobial peptide; predicted, UCLL1) | GLLRKGGEKIGEKLKKIGQKIKNFFQKLVPQPE | precursor sequence homology | 33 | 6 | 30% | 1.63 | Helix | (36) |
| Human beta defensin 3 (hBD-3, hBD3, or DEFB103, natural AMPs; human defensin, 3S=S, UCSS1a; primates, mammals, animals; ZZHh; BBBh2o; BBW; JJsn) | GIINTLQKYYCRVRGGRCAVLSCLPKEEQIGKCSTRGRKCCRRKK | skin, tonsils, oral/saliva, colonic mucosa, Homo sapiens | 45 | 11 | 33% | 2.87 | Combine Helix and Beta structure | (37) |
| Tigerinin 1 (Tigerinin-1; XXA, natural AMPs; UCSS1a; XXU; 1S=S, frog, amphibians, animals; BBMm) | FCTMIPIPRCY | skin secretions, Rana tigerina, India, Asia | 11 | 2 | 54% | 0 | Unknown | (38) |
| Buforin II (buforin 2; BF2; fragment of buforin I, UCLL1; synthetic20; VIHNN; BBN; BBL; Derivatives: buforin IIb; histonin) | TRSSRAGLQFPVGRVHRLLRK | derivative of buforin I after treatment with endoproteinase, protease digestion | 21 | 6 | 33% | 3.34 | Helix | (39) |
| LL-37 parent (LL37; cell-penetrating; natural AMPs; cathelicidin; UCLL1; human; chimpanzee; primates, mammals, animals; XXX; XXY; XXZ; BBBh2o, BBBm; BBMm, BBPP, BBN, BBL, BBrsg, JJsn) | LLGDFFRKSKEKIGKEFKRIVQRIKDFLRNLVPRTES | neutrophils, monocytes; mast cells; lymphocytes, Mesenchymal Stem Cells; islets; skin, sweat; airway surface liquid, saliva; colonic mucosa; bone marrow and testis Homo sapiens; Also Pan troglodytes | 37 | 6 | 35% | 2.99 | Helix | (40) |
| Chrysophsin-1 (CHY1; madai, natural AMPs; fish, animals; XXA; BBMm , UCLL1c) | FFGWLIKGAIHAGKAIHGLIHRRRH | the pyloric caeca and gills; Red sea bream, Chrysophrys major or Pagrus major | 25 | 9 | 48% | 1.1 | Helix | (41) |
| Caerin 1.10 (natural AMPs; UCLL1c; frog, amphibians, animals; XXA; Caerin 1.1.1, inactive; caerin 1.1.2 inactive) | GLLSVLGSVAKHVLPHVVPVIAEKL | Magnificent tree frog, Litoria splendida, Australia | 25 | 4 | 56% | -1 | Unknown | (42) |
| Citropin 1.1 (natural AMPs; UCLL1c; XXA, frog, amphibians, animals) | GLFDVIKKVASVIGGL | skin secretion, Australian blue mountains tree frog, Litoria citropa | 16 | 2 | 56% | -1.01 | Helix | (43) |
| Citropin 1.2 (natural AMPs; XXA, UCLL1c; frog, amphibians, animals) | GLFDIIKKVASVVGGL | Australian blue mountains tree frog, Litoria citropa | 16 | 2 | 56% | -1.01 | Unknown | (43) |
| Citropin 1.3 (natural AMPs; XXA, UCLL1c; frog, amphibians, animals) | GLFDIIKKVASVIGGL | Australian blue mountains tree frog, Litoria citropa | 16 | 2 | 56% | -1.06 | Unknown | (43) |
| BMAP-27 (Lys-rich; BMAP27, bovine myeloid antimicrobial peptide 27; natural AMPs; cathelicidin, cattle, ruminant, mammals, animals; UCLL1; BBN) | GRFKRFRKKFKKLFKKLSPVIPLLHLG | cattle, Bos taurus | 27 | 10 | 40% | 1.64 | Helix | (44) |
| BMAP-28 (BMAP28, bovine myeloid antimicrobial peptide 28; natural AMPs; cathelicidin-5, cattle, ruminant, mammals, animals; BBMm;ZZP; UCLL1; Derivatives: mBMAP-28 | GGLRSLGRKILRAWKKYGPIIVPIIRIG | peripheral neutrophils; cattle, Bos taurus | 28 | 7 | 42% | 0.81 | Helix | (44) |
| Penaeidin-2a (PEN2a, natural AMPs; class 1, Pro-rich domain + Cys-rich domain; UCSS1a; modular design; shrimp, Crustaceans, arthropods, invertebrates, animals) | YRGGYTGPIPRPPPIGRPPFRPVCNACYRLSVSDARNCCIKFGSCCHLVK | Penoeid shrimp, Penaeus vannamei | 50 | 7 | 36% | 1.63 | Unknown | (45) |
| PR-39 (PR39, a proline-arginine-rich peptide with 39 residues, XXA, natural AMPs; cathelicidin, pigs, mammals; animals; Pro-rich; Arg-rich; BBN) | RRRPRPPYLPRPRPPPFFPPRLPPRIPPGFPPRFPPRFP | Pig neutrophils; Sus scrofa | 39 | 11 | 20% | 3.04 | Rich | (46) |
| CPF-ST3 (XT-7; XXA, Leu-rich; natural AMPs; UCLL1c; frog, amphibians, animals, SeqAR) | GLLGPLLKIAAKVGSNLL | Diploid clawed frog Silurana tropicalis, (formerly) Xenopus tropicalis , Africa | 18 | 3 | 55% | -1.32 | Helix | (47) |
| Porcine NK-Lysin (natural AMPs; saposin-like protein, SAPLIP, pigs, mammals; animals; BBMm, BBL, ZZP; 3S=S; UCSS1a; Derivatives: NK-2; NK-18) | GLICESCRKIIQKLEDMVGPQPNEDTVTQAASRVCDKMKILRGVCKKIMRTFLRRISKDILTGKKPQAICVDIKICKE | cytotoxic T and NK cells, small intestine, domestic pig Sus scrofa | 78 | 6 | 42% | 1.92 | Helix | (48) |
| hBD-1 (Human beta-defensin 1; hBD1; natural AMPs; 3S=S; UCSS1a; primates, mammals, animals; ZZH, JJsn; XXR) | DHYNCVSSGGQCLYSACPIFTKIQGTCYRGKAKCCK | airway, hemofiltrates, urine, kidney; keratinocytes; skin; platelets; oral saliva; milk, mammary gland epithelium, colonic mucosa, Homo sapiens | 36 | 4 | 36% | 1.3 | Combine Helix and Beta structure | (49) |
| Piscidin 1 (SB piscidin-1; SB piscidin 1; SB P1; Pis-1; metallo-AMPs, natural AMPs; fish, animals; Variants: Piscidin-1N, piscidin-1H; ZZH; BBII, UCLL1) | FFHHIFRGIVHVGKTIHRLVTG | mainly mast cells, gill, skin, intestine, spleen, and anterior kidney, hybrid striped bass (Morone saxatilis x Morone chrysops); Morone saxatilis | 22 | 7 | 45% | 0.7 | Helix | (50) |
| Cupiennin 1a (Cupiennin-1a; Cu 1a; Cu-1a; Cu1a; M-ctenitoxin-Cs1a, natural AMPs; spiders, BBPP/BBII, arachnids, Chelicerata, arthropods, invertebrates, animals; XXA, UCLL1c) | GFGALFKFLAKKVAKTVAKQAAKQGAKYVVNKQME | venom, Cupiennius salei | 35 | 8 | 48% | 0.69 | Helix | (51) |
| Maximin H5 (MH5; Leu-rich; natural AMPs; UCLL1c; toad, amphibians, animals, XXA; ZZH) | ILGPVLGLVSDTLDDVLGIL | Bombina maxima, China, Asia | 20 | -2 | 55% | -1.1 | Unknown | (52) |
| Gramicidin A (gA, natural AMPs; Trp-rich; Val-rich; Leu-rich; Gram-positive bacteria, prokaryotes; nonribosomal peptide antibiotic; UCLL2b; ZZHb; ZZS; XXD5; BBMm; AMPs in use) | VGALAVVVWLWLWLW | soil bacterium, Bacillus brevis | 15 | 0 | 93% | -3.31 | Helix | (53) |
| DP1 (Ala-rich; Lys-rich; Leu-rich; synthetic20, UCLL1) | KLAKLAKKLAKLAK | KLA sequence repeats, motif-based design | 14 | 6 | 57% | 0.45 | Unknown | (54) |
| Ranalexin (natural AMPs; frog, amphibians, animals; ZZP; XXU; 1S=S, UCSS1a) | FLGGLIKIVPAMICAVTKKC | Bull frog, Rana catesbeiana, North America | 20 | 3 | 65% | -1.34 | Helix | (55) |
| Lunatusin (natural AMPs; plants, ZZHp, BWQ; unclassified) | KTCENLADTFRGPCFATSNC | Phaseolus lunatus L. (lima bean) | 20 | 0 | 40% | 2.05 | Unknown | (56) |
| Pentadactylin (Ocellatin-P1, natural AMPs; XXA; UCLL1c; frog, amphibians, animals) | GLLDTLKGAAKNVVGSLASKVMEKL | South American bullfrog, Leptodactylus pentadactylus, skin secretion, Leptodactylus labyrinthicus | 25 | 3 | 48% | 0.25 | Unknown | (57) |
| Polybia-MPI (Polybia-MP-I; Polybia-MP I; natural AMPs; insects, arthropods, invertebrates, animals, XXA; VIHNN; in vivo test) | IDWKKLLDAAKQIL | venom, social wasp, Polybia paulista | 14 | 2 | 57% | 0.64 | Helix | (58) |
| Sesquin (natural AMPs; defensins, plants, ZZHp; BWQ, unclassified) | KTCENLADTY | Seeds, Vigna sesquipedalis, ground bean | 10 | -1 | 30% | 2.49 | Unknown | (59) |
| Dybowskin-2 (Brevinin-1CEa; Brevinin-1DYe; natural AMPs; frog, amphibians, animals; XXU; 1S=S, UCSS1a) | FLIGMTQGLICLITRKC | Rana dybowskii, Rana chensinensis, Asia | 17 | 2 | 58% | -0.47 | Unknown | (60) |
| Dybowskin-3; Brevinin-2DYc; Brevinin-2RNa (natural AMPs; frog, amphibians, animals; XXU; 1S=S, UCSS1a) | GLFDVVKGVLKGVGKNVAGSLLEQLKCKLSGGC | Rana dybowskii, or Rana nigromaculata, Asia | 33 | 3 | 45% | -0.04 | Unknown | (60) |
| Brevinin-1DYa (Amurin-2c; Leu-rich; natural AMPs; frog, amphibians, animals; XXU; 1S=S, UCSS1a; Amurin-2a) | FLSLALAALPKFLCLVFKKC | Rana dybowskii, or Rana amurensis, Asia | 20 | 3 | 75% | -1.52 | Helix | (61) |
| Brevinin-1DYb (Amurin-2; Leu-rich; natural AMPs; frog, amphibians, animals; XXU; 1S=S, UCSS1a) | FLSLALAALPKLFCLIFKKC | Rana dybowskii, or Rana amurensis, Asia | 20 | 3 | 75% | -1.56 | Helix | (61) |
| Temporin-1CEb (T-1CEb, natural AMPs; Leu-rich; UCLL1c; frog, amphibians, animals; XXA; BBL) | ILPILSLIGGLLGK | Rana chensinensis, China, Asia | 14 | 2 | 57% | -2.37 | Helix | (62) |
| Pediocin PA-1/ AcH (Pediocin AcH; PedPA1, Pediocin CP2; Bacteriocin BM-1; natural AMPs; class 2a bacteriocin, bacteria, Gram-positive bacteria, prokaryotes; 2S=S, UCSS1a; Products) | KYYGNGVTCGKHSCSVDWGKATTCIINNGAMAWATGGHQGNHKC | Pediococcus acidilactici PAC-1.0; also Lactobacillus plantarum BM-1; ; lactic acid bacteria | 44 | 6 | 34% | 1.12 | Combine Helix and Beta structure | (63) |
| Maculatin 1.3 (XXA, natural AMPs; UCLL1c; frog, amphibians, animals; ZZH) | GLLGLLGSVVSHVVPAIVGHF | Litoria eucnemis, Australia | 21 | 3 | 57% | -1.77 | Unknown | (64) |
| Pardaxin 4 (Pardaxin P-4, Pardaxin P4, Pa4, natural AMPs; flat fish, animals, UCLL1) | GFFALIPKIISSPLFKTLLSAVGSALSSSGGQE | Red Sea moses sole, Pardachirus marmoratus | 33 | 1 | 45% | -0.35 | Helix | (65) |
| GF-17 (GF17; FK-16, XXA; ZZHs, synthetic20, fragment of LL-37, UCLL1; engineered: GF17d3; GF-17d3; 17BIPHE2; merecidin) | GFKRIVQRIKDFLRNLV | Derivative of LL-37; NMR-based discovery; Sequence truncation. human cathelicidin analog, animal-derived, natural derivative | 17 | 5 | 47% | 2.47 | Helix | (66) |
| Decoralin (Dec-NH2; Leu-rich; natural AMPs; insects, arthropods, invertebrates, animals, UCLL1a) | SLLSLIRKLIT | venom, solitary eumenine wasp, Oreumenes decoratus | 11 | 2 | 54% | 0.02 | Helix | (67) |
| Maculatin 1.4 (XXA, Leu-rich; natural AMPs; UCLL1c; frog, amphibians, animals) | GLLGLLGSVVSHVLPAITQHL | Litoria eucnemis, Australia | 21 | 3 | 52% | -1.28 | Unknown | (64) |
| NRC-03 (NRC-3; XXA, natural AMPs; fish, animals; UCLL1c) | GRRKRKWLRRIGKGVKIIGGAALDHL | winter flounder 1a-1, Pleuronectes americanus | 26 | 9 | 38% | 2.53 | Unknown | (68) |
| Human lactoferricin (metallo-AMPs, natural AMPs; primates, mammals, animals; BBII, XXA; 2S=S; UCLL1c) | GRRRRSVQWCAVSQPEATKCFQWQRNMRKVRGPPVSCIKRDSPIQCIQA | milk, Homo sapiens | 49 | 10 | 36% | 3.14 | Helix | (69) |
| Bombinin H2 (XXA, Leu-rich; natural AMPs; UCLL1c; toad, amphibians, animals) | LIGPVLGLVGSALGGLLKKI | Bombina variegata, Europe | 20 | 3 | 55% | -1.97 | Helix | (70) |
| hepcidin TH1-5 (Cys-rich 36%; 4S=S, natural AMPs; UCSS1a; fish, animals; TH2-2, inactive) | GIKCRFCCGCCTPGICGVCCRF | tilapia, Oreochromis mossambicus | 22 | 3 | 59% | 0.18 | Bridge | (71) |
| hepcidin TH2-3 (Cys-rich 30%; 4S=S, natural AMPs; UCSS1a; fish, animals) | QSHLSLCRWCCNCCRSNKGC | tilapia, Oreochromis mossambicus | 20 | 3 | 45% | 2.41 | Unknown | (71) |
| Temporin-1Oc (XXA; Leu-rich; natural AMPs; UCLL1c; frog, amphibians, animals) | FLPLLASLFSRLF | Rana Ornativentris, Asia | 13 | 2 | 69% | -1.04 | Unknown | (72) |
| Maximin H1 (XXA, natural AMPs; UCLL1c; toad, amphibians, animals) | ILGPVISTIGGVLGGLLKNL | Bombina maxima, China, Asia | 20 | 2 | 50% | -1.69 | Unknown | (9) |
| Brevinin-ALb (Brevinin ALb, natural AMPs; frog, amphibians, animals; XXU; 1S=S, UCSS1a) | FLPLAVSLAANFLPKLFCKITKKC | Amolops loloensis, China, Asia | 24 | 4 | 62% | -0.65 | Unknown | (73) |
| Temporin-ALa (Temporin ALa, XXA; Leu-rich; natural AMPs; UCLL1c; frog, amphibians, animals) | FLPIVGKLLSGLSGLL | the rufous-spotted torrent frog, Amolops loloensis, China, Asia | 16 | 2 | 56% | -1.99 | Unknown | (73) |
| Temporin 1OLa (XXA, Temporin-1OLa, Leu-rich; natural AMPs; UCLL1c; frog, amphibians, animals. More AMPs?) | FLPFLKSILGKIL | Florida bog frog, Rana okaloosae, North America | 13 | 3 | 61% | -1.68 | Helix | (74) |
| Brevinin-1BYa (Brevinin 1BYa, natural AMPs; frog, amphibians, animals; XXU; 1S=S, UCSS1a) | FLPILASLAAKFGPKLFCLVTKKC | skin secretions, the foothill yellow-legged frog, Rana boylii, North America | 24 | 4 | 62% | -0.96 | Helix | (75) |
| Pexiganan acetate (MSI 78, MSI-78; Lys-rich; analog of magainin, cytolex, loxilex; Synthetic20, XXA, UCLL1c; BBMm; clinical trials) | GIGKFLKKAKKFGKAFVKILKK | Engineered | 22 | 10 | 45% | 0.49 | Helix | (76) |
| Dermaseptin-L1 (Dermaseptin L1, DRS-L1; UCLL1; natural AMPs; frog, amphibians, animals) | GLWSKIKEAAKAAGKAALNAVTGLVNQGDQPS | Hylomantis lemur (Hylidae: Phyllomedusinae), South America | 32 | 2 | 43% | 0.77 | Unknown | (77) |
| Phylloseptin-L1 (PLS-L1; Medusin-AC; UCLL1c; Leu-rich; natural AMPs; frog, amphibians, animals; XXA) | LLGMIPLAISAISALSKL | Hylomantis lemur (Hylidae: Phyllomedusinae, South America; also skin secretion, red-eyed leaf frog, Agalychnis callidryas, Central America | 18 | 2 | 66% | -1.79 | Unknown | (77) |
| NaD1 (N. alata defensin 1, Na-D1; natural AMPs; defensins; 4s=s; UCSS1a; ZZI; plants; BBBH2o) | RECKTESNTFPGICITKPPCRKACISEKFTDGHCSKILRRCLCTKPC | ornamental tobacco flowers, Nicotiana alata | 47 | 6 | 36% | 2.27 | Combine Helix and Beta structure | (78) |
| TPP3 (natural AMPs; defensins; 4S=S; UCSS1a; plants; BBMm) | QICKAPSQTFPGLCFMDSSCRKYCIKEKFTGGHCSKLQRKCLCTKPC | tomato, Lycopersicon esculentum | 47 | 7 | 38% | 1.62 | Combine Helix and Beta structure | (79) |
| Latarcin 3a (Latarcin-3a, Ltc3a, Ltc 3a, La47, XXA, Lys-rich; BBMm; natural AMPs; spiders, arachnids, Chelicerata, arthropods, invertebrates, animals; VIHNN; UCLL1c) | SWKSMAKKLKEYMEKLKQRA | venom, Lachesana tarabaevi, Central Asia | 20 | 6 | 35% | 2.69 | Helix | (80) |
| Varv peptide A (Varv A, kalata S; natural non-AMPs; cyclotides; flowering seed plants; XXC; 3S=S, UCBB1aB) | GLPVCGETCVGGTCNTPGCSCSWPVCTRN | Viola arvensis,Viola odorata, Viola tricolor, Viola baoshanensis, Viola yedoensis, and Viola biflora | 29 | 0 | 37% | 0.7 | Bridge | (81) |
| Varv peptide F (Varv F, natural non-AMPs; cyclotides; flowering seed plants; XXC; 3S=S, UCBB1aB; plants) | GVPICGETCTLGTCYTAGCSCSWPVCTRN | Viola arvensis | 29 | 0 | 41% | 0.5 | Beta | (82) |
| Cycloviolacin O2 (CyO2; natural AMPs; cyclotides; flowering seed plants; XXC; 3S=S, UCBB1aB; ZZP) | GIPCGESCVWIPCISSAIGCSCKSKVCYRN | Viola odorata and Viola japonica | 30 | 2 | 46% | 0.47 | Bridge | (83) |
| Vibi E (natural non-AMPs; cyclotides; natural, plants; 3S=S, UCBB1ab; XXC) | GIPCAESCVWIPCTVTALIGCGCSNKVCYN | alpine violet Viola biflora | 30 | 0 | 53% | -0.35 | Bridge | (84) |
| Vibi G (natural non-AMPs; cyclotides; plants;3S=S, UCBB1ab; XXC) | GTFPCGESCVFIPCLTSAIGCSCKSKVCYKN | alpine violet Viola biflora | 31 | 2 | 45% | 0.25 | Bridge | (84) |
| Vibi H (natural non-AMPs; cyclotides; plants; 3S=S, UCBB1ab; XXC) | GLLPCAESCVYIPCLTTVIGCSCKSKVCYKN | alpine violet Viola biflora | 31 | 2 | 48% | -0.07 | Bridge | (84) |
| Human granulysin (GNLY, huGran, NKG5; formerly 519; a saposin-like protein, SAPLIP; 3S=S; natural AMPs; UCSS1a; primates, mammals, animals; ZZP; VIHNN; BBMm; BBL; JJsn; Derivatives: G13) | GRDYRTCLTIVQKLKKMVDKPTQRSVSNAATRVCRTGRSRWRDVCRNFMRRYQSRVTQGLVAGETAQQICEDLR | NK cells; T lymphocytes; Homo sapiens | 74 | 11 | 33% | 3.5 | Helix | (85) |
| Microcin E492 (MccE492, metallo-AMPs, natural AMPs; class 2b microcins, the siderophore-mocrocin family; bacteriocin, Gram-negative bacteria, prokaryotes; BBPP; BBII, XXB; BBBh2o; Variants: u-Mcc492) | GETDPNTQLLNDLGNNMAWGAALGAPGGLGSAALGAAGGALQTVGQGLIDHGPVNVFIPVLIGPSWNGSGSGYNSATSSSGSGS | Klebsiella pneumoniae RYC492; human microbiota:gut, symbiont bacteria | 84 | -4 | 35% | 0.28 | Unknown | (86) |
| Ascaphin-8 (XXA, natural AMPs; UCLL1c; frog, amphibians, animals; BBMm; SeqAR; ZZH; Derivatives: A8-2-o and A8-4-Dp; stapled peptides) | GFKDLLKGAAKALVKTVLF | Coastal Tailed Frog, Ascaphus truei, Pacific Northwest, USA, North America | 19 | 4 | 57% | -0.39 | Helix | (87) |
| Cathelicidin-BF (BF-30; Lys-rich; UCLL1, natural AMPs; cathelicidin-WA; snake cathelicidin, reptiles, animals; Derivatives: BF-15; Cbf-K16) | KFFRKLKKSVKKRAKEFFKKPRVIGVSIPF | venom, banded krait, [Bungarus fasciatus](https://en.wikipedia.org/wiki/Banded_krait) | 30 | 11 | 40% | 2.12 | Helix | (88) |
| Mouse beta-defensin-14 (mBD-14, Defb14, natural AMPs; UCSS1a; mouse, rodents, mammals, animals; 3S=S) | FLPKTLRKFFCRIRGGRCAVLNCLGKEEQIGRCSNSGRKCCRKKK | spleen, colon, and tissues of the upper and lower respiratory tract, Mus musculus | 45 | 12 | 37% | 2.85 | Bridge | (89) |
| CM4 (ABP-CM4, UCLL1; natural AMPs; insects, arthropods, invertebrates, animals; BBMm; BBN) | RWKIFKKIEKVGQNIRDGIVKAGPAVAVVGQAATI | hemolymph, Chinese silkworm, Bombyx mori | 35 | 5 | 48% | 0.87 | Helix | (90) |
| Viscotoxin A3 (VtA3, thionins; natural AMPs; UCSS1a; plants, BBMm; 3S=S) | KSCCPNTTGRNIYNACRLTGAPRPTCAKLSGCKIISGSTCPSDYPK | the European mistletoe, Viscum album L | 46 | 6 | 30% | 1.83 | Combine Helix and Beta structure | (91) |
| Viscotoxin 1-Ps (Vt1-Ps, thionins; natural non-AMPs; UCSS1a; 3S=S, plants) | KSCCPNTTGRNIYNTCRFGGGSREVCARISGCKIISASTCPSDYPK | the European mistletoe, Viscum album L | 46 | 5 | 30% | 2.17 | Unknown | (92) |
| Viscotoxin A1 (VtA1, thionins; natural non-AMPs; UCSS1a; 3S=S, plants) | KSCCPNTTGRNIYNTCRLTGSSRETCAKLSGCKIISASTCPSNYPK | seeds, Viscum album L | 46 | 6 | 28% | 2.19 | Combine Helix and Beta structure | (92) |
| Viscotoxin C (Viscotoxin C1, VtC1, thionins; natural non-AMPs; UCSS1a; 3S=S, plants) | KSCCPNTTGRNIYNTCRFAGGSRERCAKLSGCKIISASTCPSDYPK | the Asiatic Viscum album ssp Coloratum ohwi | 46 | 6 | 30% | 2.36 | Combine Helix and Beta structure | (92) |
| Viscotoxin A2 (VtA2, thionins; natural non-AMPs; UCSS1a; 3S=S, plants) | KSCCPNTTGRNIYNTCRFGGGSRQVCASLSGCKIISASTCPSDYPK | Viscum album L | 46 | 5 | 30% | 1.89 | Combine Helix and Beta structure | (92) |
| Viscotoxin B (VtB, thionins; natural non-AMPs; UCSS1a; 3S=S, plants) | KSCCPNTTGRNIYNTCRLGGGSRERCASLSGCKIISASTCPSDYPK | Viscum album L | 46 | 5 | 28% | 2.29 | Combine Helix and Beta structure | (93) |
| Pep27 (natural AMPs; bacteriocin; Gram-positive bacteria, prokaryotes; Derivatives) | MRKEFHNVLSSGQLLADKRPARDYNRK | Streptococcus pneumoniae | 27 | 4 | 29% | 3.78 | Helix | (94) |
| Viscotoxin B2 (VtB2, thionins; natural non-AMPs; UCSS1a; 3S=S, plants) | KSCCKNTTGRNIYNTCRFAGGSRERCAKLSGCKIISASTCPSDYPK | Viscum coloratum (Kom.) Nakai | 46 | 7 | 30% | 2.48 | Combine Helix and Beta structure | (95) |
| Epinecidin-1 (Epi-1; piscidin, natural AMPs; fish, animals; XXA; BBMm; Variant: Ac-Var-1) | GFIFHIIKGLFHAGKMIHGLV | orange-spotted grouper, Epinephelus coioides | 21 | 6 | 57% | -1.2 | Unknown | (96) |
| Cn-AMP1 (CnAMP1, XXA; C. nucifera antimicrobial peptide 1, natural AMPs; plants) | SVAGRAQGM | green coconut water, Cocos nucifera | 9 | 2 | 44% | 1.32 | Helix | (97) |
| Cn-AMP2 (XXA; C. nucifera antimicrobial peptide 2, natural AMPs; plants) | TESYFVFSVGM | green coconut water, Cocos nucife | 11 | 0 | 45% | -0.09 | Unknown | (97) |
| Brevinin-1BLa (natural AMPs; frog, amphibians, animals; XXU; 1S=S, UCSS1a) | FLPAIVGAAAKFLPKIFCAISKKC | North America, leopard frog, Lithobates blairi | 24 | 4 | 66% | -1.02 | Unknown | (98) |
| Brevinin-1BLb (natural AMPs; frog, amphibians, animals; XXU; 1S=S, UCSS1a) | FLPIIAGVAAKVLPKIFCAISKKC | North America, leopard frog, Lithobates blairi | 24 | 4 | 66% | -1.19 | Unknown | (98) |
| Brevinin-1BLc (natural AMPs; frog, amphibians, animals; XXU; 1S=S, UCSS1a) | FLPIIAGIAAKFLPKIFCTISKKC | North America, leopard frog, Lithobates blairi | 24 | 4 | 62% | -1 | Unknown | (98) |
| Brevinin-1Ya (natural AMPs; frog, amphibians, animals; XXU; 1S=S, UCSS1a) | FLPVIAGVAANFLPKLFCAISKKC | North America, leopard frog, Lithobates yavapaiensis | 24 | 3 | 66% | -1.06 | Unknown | (98) |
| Brevinin-1Yb (natural AMPs; frog, amphibians, animals; XXU; 1S=S, UCSS1a) | FLPIIAGAAAKVVQKIFCAISKKC | North America, leopard frog, Lithobates yavapaiensis | 24 | 4 | 66% | -0.83 | Unknown | (98) |
| Brevinin-1Yc (natural AMPs; frog, amphibians, animals; XXU; 1S=S, UCSS1a) | FLPIIAGAAAKVVEKIFCAISKKC(98) | North America, leopard frog, Lithobates yavapaiensis | 24 | 3 | 66% | -0.78 | Unknown | (98) |
| Ranatuerin-2Ya (natural AMPs; frog, amphibians, animals; XXU; 1S=S, UCSS1a) | GLMDTIKGVAKTVAASWLDKLKCKITGC | North America, leopard frog, Lithobates yavapaiensis | 28 | 3 | 50% | 0.29 | Unknown | (98) |
| Alyteserin-2a (XXA; UCLL1c; Leu-rich; natural AMPs; toad, amphibians, animals) | ILGKLLSTAAGLLSNL | the European midwife toad, Alytes obstetricans | 16 | 2 | 56% | -1.14 | Helix | (99) |
| Lasioglossin LL-I (XXA; Lys-rich; natural AMPs; insects, arthropods, invertebrates, animals) | VNWKKVLGKIIKVAK | the Eusocial Bee Lasioglossum laticeps | 15 | 6 | 53% | 0.16 | Helix | (100) |
| Lasioglossin LL-II (XXA; Lys-rich; natural AMPs; insects, arthropods, invertebrates, animals; BBN) | VNWKKILGKIIKVAK | the Eusocial Bee Lasioglossum laticeps | 15 | 6 | 53% | 0.1 | Helix | (100) |
| Lasioglossin LL-III (XXA; Lasioglossin III, LLIII, Lasio-III, Lys-rich; natural AMPs; insects, arthropods, invertebrates, animals; Derivative: LLIII-D; stapled peptides) | VNWKKILGKIIKVVK | the Eusocial Bee Lasioglossum laticeps | 15 | 6 | 53% | -0.04 | Helix | (100) |
| CGA-N46 (natural AMPs; A fragment of human chromogranin A, neuropeptide; UCLL1. Derivatives: CGA-N16, CGA-N15, CGA-N12, and CGA-N8) | PMPVSQECFETLRGHERILSILRHQNLLKELQDLALQGAKERAHQQ | Homo sapiens | 46 | 3 | 36% | 2.38 | Helix | (101) |
| Imcroporin (natural AMPs; scorpions, arachnids, Chelicerata, arthropods, invertebrates, animals; XXA, UCLL1c) | FFSLLPSLIGGLVSAIK | Isometrus maculates | 17 | 2 | 58% | -1.61 | Unknown | (102) |
| EP3 (XXE; earthworm peptide, natural AMPs; invertebrate, animals) | AMVGT | earthworm, Eisenia fetida | 5 | -1 | 60% | -1.31 | Unknown | (103) |
| ChBac3.4 (XXA; Arg-rich, natural AMPs; Pro-rich cathelicidin; goat, ruminant, mammals; animals) | RFRLPFRRPPIRIHPPPFYPPFRRFL | goat, Capra hirca | 26 | 8 | 34% | 2.87 | Rich | (104) |
| Temporin-SHf (Phe-rich; natural AMPs; frog, amphibians, animals; XXA; UCLL1c) | FFFLSRIF | Sahara Frog, Pelophylax saharicus, Africa | 8 | 2 | 75% | -0.42 | Helix | (105) |
| Drosophila defensin (natural AMPs; insects, UCSS1a; 3S=S, arthropods, invertebrates, animals) | ATCDLLSKWNWNHTACAGHCIAKGFKGGYCNDKAVCVCRN | fruit fly, Drosophila melanogaster | 40 | 5 | 47% | 1.2 | Bridge | (106) |
| P-18 (Lys-rich; synthetic20; UCLL1; Derivatives: S9-P18; L9-P18; BBMm) | KWKLFKKIPKFLHLAKKF | Sequence reverse, shuffling | 18 | 7 | 50% | 0.59 | Unknown | (107) |
| Crotamine (Crot; 3S=S; defensin-like toxin; UCSS1a; cell-penetrating; natural AMPs; South American rattlesnake, reptiles, animals; ZZP; Derivatives: CyLoP-1) | YKQCHKKGGHCFPKEKICLPPSSDFGKMDCRWRWKCCKKGSG | venom, Crotalus durissus terrificus | 42 | 10 | 30% | 2.23 | Combine Helix and Beta structure | (108) |
| Cliotide T1 (cT1; natural AMPs; cyclotides; XXC; 3S=S, UCBB1ab; Fabaceae, plants; Other possible AMPs? Cliotide T5, Cliotide T6, Cliotide T8, Cliotide T9, Cliotide T11) | GIPCGESCVFIPCITGAIGCSCKSKVCYRN | Clitoria ternatea | 30 | 2 | 46% | 0.27 | Unknown | (109) |
| Cliotide T2 (cT2; natural non-AMPs; cyclotides; XXC; 3S=S, UCBB1ab; Fabaceae, plants; Others: cT5, cT6, cT7, cT8, cT9, cT10, cT11, cT12) | GEFLKCGESCVQGECYTPGCSCDWPICKKN | Clitoria ternatea | 30 | -1 | 36% | 1.22 | Unknown | (109) |
| Cliotide T3 (cT3; natural non-AMPs; cyclotides; XXC; 3S=S, UCBB1ab; Fabaceae, plants) | GLPTCGETCTLGTCYVPDCSCSWPICMKN | Clitoria ternatea | 29 | -1 | 41% | 0.37 | Unknown | (109) |
| Cliotide T4 (cT4; natural AMPs; cyclotides; XXC; 3S=S, UCBB1ab; Fabaceae, plants) | GIPCGESCVFIPCITAAIGCSCKSKVCYRN | Clitoria ternatea | 30 | 2 | 50% | 0.25 | Unknown | (109) |
| Vaby A (natural non-AMPs; cyclotides; plants; 3S=S, UCBB1ab; XXC; other AMPs?: Vaby B; VabyC; and VabyE) | GLPVCGETCAGGTCNTPGCSCSWPICTRN | Africa, the Ethiopian highlands, Viola abyssinica | 29 | 0 | 37% | 0.74 | Unknown | (110) |
| Vaby D (natural non-AMPs; cyclotides; plants; 3S=S; XXC, UCBB1ab;) | GLPVCGETCFGGTCNTPGCTCDPWPVCTRN | Africa, the Ethiopian highlands, Viola abyssinica | 30 | -1 | 36% | 0.86 | Unknown | (110) |
| Temporin-1CEa (Ile-rich; UCLL1c; natural AMPs; frog, amphibians, animals; XXA) | FVDLKKIANIINSIF | Chinese brown frog, skin secretions, Rana chensinensis, China, Asia | 15 | 2 | 60% | 0 | Helix | (62) |
| Cr-ACP1 (C. revoluta anticancer peptide 1, natural AMPs; plants) | AWKLFDDGV | seeds, Cycas revoluta | 9 | -1 | 55% | 0.66 | Unknown | (111) |
| Viba 15 (natural non-AMPs; cyclotides; plants; 3S=S, UCBB1ab; XXC) | GLPVCGETCVGGTCNTPGCACSWPVCTRN | Viola philippica | 29 | 0 | 41% | 0.52 | Bridge | (112) |
| Viba17 (natural non-AMPs; cyclotides; plants; 3S=S, UCBB1ab; XXC) | GLPVCGETCVGGTCNTPGCGCSWPVCTRN | Viola philippica | 29 | 0 | 37% | 0.55 | Bridge | (112) |
| Viphi A (natural non-AMPs; cyclotides; plants; 3S=S, UCBB1ab; XXC) | GSIPCGESCVFIPCISSVIGCACKSKVCYKN | Viola philippica | 31 | 2 | 48% | 0 | Bridge | (112) |
| Viphi D (natural non-AMPs; cyclotides; plants; 3S=S, UCBB1ab; XXC) | GIPCGESCVFIPCISSVIGCSCSSKVCYRN | Viola philippica | 30 | 1 | 46% | 0.3 | Bridge | (112) |
| Viphi E (natural non-AMPs; cyclotides; plants; 3S=S, UCBB1ab; XXC) | GSIPCGESCVFIPCISAVIGCSCSNKVCYKN | Viola philippica | 31 | 1 | 48% | 0.03 | Bridge | (112) |
| Viphi F (natural non-AMPs; cyclotides; plants; 3S=S, UCBB1ab; XXC) | GSIPCGESCVFIPCISAIIGCSCSSKVCYKN | Viola philippica | 31 | 1 | 48% | -0.09 | Bridge | (112) |
| Viphi G (natural non-AMPs; cyclotides; plants; 3S=S, UCBB1ab; XXC) | GSIPCEGSCVFIPCISAIIGCSCSNKVCYKN | Viola philippica | 31 | 1 | 48% | 0.01 | Bridge | (112) |
| Mram 8 (natural non-AMPs; cyclotides; plants; 3S=S, UCBB1ab; XXC) | GIPCGESCVFIPCLTSAIDCSCKSKVCYRN | Viola philippica | 30 | 1 | 46% | 0.74 | Bridge | (112) |
| Halictine 1 (Halictine-1; HAL-1; XXA; natural AMPs; insects, arthropods, invertebrates, animals) | GMWSKILGHLIR | venom, the eusocial bee, Halictus sexcinctus | 12 | 4 | 50% | 0.19 | Helix | (113) |
| Halictine 2 (Halictine-2, HAL-2; XXA; natural AMPs; insects, arthropods, invertebrates, animals) | GKWMSLLKHILK | venom, the eusocial bee, Halictus sexcinctus | 12 | 5 | 50% | -0.04 | Helix | (113) |
| Macropin 1 (Mac1, MAC-1, Leu-rich; natural AMPs; insects, arthropods, invertebrates, animals, XXA) | GFGMALKLLKKVL | venom, the solitary bee Macropis fulvipes | 13 | 4 | 61% | -1.23 | Helix | (114) |
| Macropin 2 (Mac2, natural AMPs; insects, arthropods, invertebrates, animals, XXA) | GTGLPMSERRKIMLMMR | venom, the solitary bee Macropis fulvipes | 17 | 4 | 41% | 2.17 | Unknown | (114) |
| Hymenochirin-1B (Hym-1B; natural AMPs; frog, amphibians, animals; XXA; UCLL1c) | IKLSPETKDNLKKVLKGAIKGAIAVAKMV | Congo dwarf clawed frog, African dwarf frog, Hymenochirus boettgeri, Africa | 29 | 6 | 48% | 0.47 | Unknown | (115) |
| BmKn2 (BmKn-2, natural AMPs; scorpions, arachnids, Chelicerata, arthropods, invertebrates, animals; XXA, UCLL1c; derivatives: cyclic BmKn2) | FIGAIARLLSKIF | venom, Buthus martensii Karsch | 13 | 3 | 69% | -0.86 | Helix | (116) |
| Psyle A (natural non-AMPs; cyclotides; plants; 3S=S, UCBB1ab; XXC. Other AMPs? psyle B, D, F ) | GIACGESCVFLGCFIPGCSCKSKVCYFN | Psychotria leptothyrsa | 28 | 1 | 53% | -0.36 | Bridge | (117) |
| Psyle E (natural non-AMPs; cyclotides; plants; 3S=S, UCBB1ab; XXC) | GVIPCGESCVFIPCISSVLGCSCKNKVCYRD | Psychotria leptothyrsa | 31 | 1 | 48% | 0.4 | Bridge | (117) |
| Psyle C (natural non-AMPs; uncyclo-tides; UCSS1a; 3S=S, plants) | KLCGETCFKFKCYTPGCSCSYPFCK | Psychotria leptothyrsa | 25 | 3 | 40% | 0.71 | Bridge | (117) |
| ChaC1 (chassatide C1, natural non-AMPs; cyclotides; plants; 3S=S, UCBB1ab; More AMPs? chaC3, chaC5, chaC6, chaC9, chaC12-18) | GDACGETCFTGICFTAGCSCNPWPTCTRN | hybrid peptide of melittin and protamine. | 29 | -1 | 41% | 1.09 | Bridge | (118) |
| ChaC2 (chassatide C2, natural non-AMPs; cyclotides; flowering seed plants; 3S=S, UCBB1ab; XXC; Variants: chaC2A, XXO) | GIPCAESCVWIPPCTITALMGCSCKNNVCYNN | Chassalia chartacea (or Chassalia curviflora) | 32 | 0 | 50% | 0.16 | Bridge | (118) |
| ChaC4 (chassatide C4, natural non-AMPs; cyclotides; flowering seed plants; 3S=S, UCBB1ab; XXC) | GASCGETCFTGICFTAGCSCNPWPTCTRN | Chassalia chartacea (or Chassalia curviflora) | 29 | 0 | 41% | 0.91 | Bridge | (118) |
| ChaC7 (chassatide C7, natural AMPs; uncyclo-tides; UCSS1a; 3S=S, flowering seed plants) | IPCGESCVWIPCITAIAGCSCKNKVCYT | Chassalia chartacea (or Chassalia curviflora) | 28 | 1 | 53% | -0.23 | Bridge | (118) |
| ChaC8 (chassatide C8, natural AMPs; uncyclo-tides; UCSS1a; 3S=S, flowering seed plants) | AIPCGESCVWIPCISTVIGCSCSNKVCYR | Chassalia chartacea (or Chassalia curviflora) | 29 | 1 | 51% | 0.16 | Bridge | (118) |
| ChaC10 (chassatide C10, natural non-AMPs; cyclotides; flowering seed plants; 3S=S, UCBB1ab; XXC) | GEYCGESCYLIPCFTPGCYCVSRQCVNKN | Chassalia chartacea (or Chassalia curviflora) | 29 | 0 | 37% | 1.07 | Bridge | (118) |
| ChaC11 (chassatide C11, natural AMPs; uncyclo-tides; UCSS1a; 3S=S, flowering seed plants. Variants: chaC11A, XXO) | IPCGESCVWIPCISGMFGCSCKDKVCYS | Chassalia chartacea (or Chassalia curviflora) | 28 | 0 | 50% | -0.02 | Bridge | (118) |
| CCL21 (SLC, cytokine, CC family; kinocidins; antimicrobial protein; UCSS1a; 3S=S, natural AMPs; human, primates, mammals, animals) | SDGGAQDCCLKYSQRKIPAKVVRSYRKQEPSLGCSIPAILFLPRKRSQAELCADPKELWVQQLMQHLDKTPSPQKPAQGCRKDRGASKTGKKGKGSKGCKRTERSQTPKGP | Homo sapiens | 111 | 17 | 27% | 2.65 | Combine Helix and Beta structure | (119) |
| Esculentin-2CHa (natural AMPs; frog, amphibians, animals; XXU; 1S=S, UCSS1a) | GFSSIFRGVAKFASKGLGKDLAKLGVDLVACKISKQC | skin secretions, Chiricahua leopard frog, Lithobates chiricahuensis, Arizona, USA, North America | 37 | 5 | 48% | 0.53 | Unknown | (120) |
| TP3 (Tilapia piscidin 3; His-rich; natural AMPs; fish, animals; probably inactive: TP1, TP2, and TP5 MIC >17.64-23.57 ug/ml) | FIHHIIGGLFSVGKHIHSLIHGH | Nile Tilapia, Oreochromis niloticus | 23 | 7 | 43% | -0.34 | Unknown | (121) |
| TP4 (Tilapia piscidin 4; Oreoch-2; MSP-4; natural AMPs; fish, animals; BBL; Derivative: TP4-3; stapled peptides) | FIHHIIGGLFSAGKAIHRLIRRRRR | gills, Nile Tilapia, Oreochromis niloticus | 25 | 10 | 44% | 2.62 | Helix | (121) |
| Pis1 (Gaduscidin-1, GAD-1; piscidin; His-rich classic; Ile-rich; metallo-AMPs; natural AMPs; fish, animals; XXA, UCLL1c, BBII. inactive variant: pis2-beta) | FIHHIIGWISHGVRAIHRAIH | Atlantic cod, Gadus morhua L. | 21 | 8 | 52% | 0.57 | Helix | (122) |
| Pis2 (piscidin; His-rich classic; natural AMPs; fish, animals, UCLL1: Variants: GAD-2) | FLHHIVGLIHHGLSLFGDRAD | Atlantic cod, Gadus morhua L. | 21 | 3 | 47% | 0.48 | Helix | (122) |
| Temporin-La (Leu-rich; natural AMPs; frog, amphibians, animals; XXA; UCLL1c) | LLRHVVKILEKYL | Skin,The American bullfrog, Lithobates Catesbeianus, North America | 13 | 4 | 53% | 0.38 | Unknown | (123) |
| Palustrin-Ca (XXU; 1S=S, UCSS1a; natural AMPs; frog, amphibians, animals) | GFLDIIKDTGKEFAVKILNNLKCKLAGGCPP | Skin,The American bullfrog, Lithobates Catesbeianus, North America | 31 | 2 | 45% | 0.43 | Unknown | (123) |
| C18G (Leu-rich, Lys-rich; Synthetic20, UCLL1a; BBMm; C18X) | ALYKKLLKKLLKSAKKLG | derivative of the carboxyl terminus of human platelet factor IV, animal-derived, natural derivative | 18 | 7 | 44% | 0.46 | Helix | (124) |
| VmCT1 (natural AMPs; scorpions, arachnids, Chelicerata, arthropods, invertebrates, animals; XXA, UCLL1c) | FLGALWNVAKSVF | Vaejovis mexicanus smithi | 13 | 2 | 69% | -1.16 | Helix | (125) |
| Lycosin-I (XXA; Lys-rich; natural AMPs; spiders, arachnids, Chelicerata, arthropods, invertebrates, animals; BBMm; UCLL1c) | RKGWFKAMKSIAKFIAKEKLKEHL | Venom, Lycosa singorensis | 24 | 8 | 45% | 1.61 | Helix | (126) |
| TsAP-S1 (Lys-rich; synthetic20; XXA, UCLL1c; TsAP-1: inactive) | FLSLIPKLVKKIIKAFK | The inactive natural peptide TsAP-1 was made active by increasing 4 lysines and one leucine, template-based design | 17 | 6 | 59% | -0.59 | Unknown | (127) |
| TsAP-2 (NDBP-4.23, T. serrulatus antimicrobial peptide 2; natural AMPs; scorpions, arachnids, Chelicerata, arthropods, invertebrates, animals; UCLL1a; Derivative: TsAP-S2) | FLGMIPGLIGGLISAFK | venom, the Brazilian yellow scorpion, Tityus serrulatus, also Turrilites costatus; Tityus obscurus, South America | 17 | 1 | 58% | -2.02 | Unknown | (127) |
| Chensinin-1b (C1b, UCLL1; Arg-rich; synthetic20; BBL) | SKVWRHWRRFWHRAHRLH | Designed based on Chensinin-1 | 18 | 10 | 39% | 4.52 | Helix | (128) |
| Temporin-1RNa (Leu-rich; natural AMPs; frog, amphibians, animals; XXA; UCLL1c) | ILPIRSLIKKLL | the black-spotted frog, Rana nigromaculata ,Northeastern China, Asia | 12 | 4 | 58% | -0.41 | Helix | (129) |
| Temporin-1RNb (Leu-rich; natural AMPs; frog, amphibians, animals; XXA; UCLL1c) | FLPLKKLRFGLL | the black-spotted frog, Rana nigromaculata ,Northeastern China, Asia | 12 | 4 | 58% | -0.45 | Helix | (129) |
| Hymenochirin-1Pa (natural AMPs; frog, amphibians, animals; XXA; UCLL1c) | LKLSPKTKDTLKKVLKGAIKGAIAIASMA | skin secretions, Merlin's clawed frog, Pseudhymenochirus merlini, Africa | 29 | 7 | 48% | 0.26 | Helix | (130) |
| Pseudhymenochirin-1Pb (Ps-1Pb; natural AMPs; frog, amphibians, animals; UCLL1) | IKIPSFFRNILKKVGKEAVSLIAGALKQS | skin secretions, Merlin's clawed frog, Pseudhymenochirus merlini, Africa | 29 | 5 | 48% | 0.55 | Helix | (130) |
| Pseudhymenochirin-2Pa (Ps-2Pa; natural AMPs; frog, amphibians, animals; UCLL1) | GIFPIFAKLLGKVIKVASSLISKGRTE | skin secretions, Merlin's clawed frog, Pseudhymenochirus merlini, Africa | 27 | 4 | 48% | 0.06 | Helix | (130) |
| Cliotide T7 (CT7; Cter R; natural AMPs; cyclotides; XXC; 3S=S, UCBB1ab; Fabaceae, plants) | GIPCGESCVFIPCTVTALLGCSCKDKVCYKN | Clitoria ternatea | 31 | 1 | 48% | 0.12 | Unknown | (109) |
| Cliotide T10 (CT10; Cter B; natural AMPs; cyclotides; XXC; 3S=S, UCBB1ab; Fabaceae, plants) | GVPCAESCVWIPCTVTALLGCSCKDKVCYLN | Clitoria ternatea | 31 | 0 | 54% | -0.19 | Unknown | (109) |
| Cliotide T12 (CT12; natural non-AMPs; cyclotides; XXC; 3S=S, UCBB1ab; Fabaceae, plants) | GIPCGESCVYIPCTVTALLGCSCKDKVCYKN | Clitoria ternatea | 31 | 1 | 45% | 0.22 | Unknown | (109) |
| Cliotide T19 (CT19; natural AMPs; cyclotides; XXC; 3S=S, UCBB1ab; Fabaceae, plants) | GSVIKCGESCLLGKCYTPGCTCSRPICKKD | Clitoria ternatea | 30 | 3 | 36% | 1.09 | Unknown | (131) |
| Lunasin (natural non-AMPs; plants) | SKWQHQQDSCRKQLQGVNLTPCEKHIMEKIQGRGDDDDDDDDD | Glycine max | 43 | -4 | 20% | 4.13 | Unknown | (132) |
| PaDef (P. americana defensin; natural AMPs; plants; UCSS1a; 4S=S) | CETPSKHFNGLCIRSSNCASVCHGEHFTDGRCQGVRRRCMCLKPC | Avocado Fruit, [Persea americana](http://www.flmnh.ufl.edu/soltislab/fgp_education/persea.html) var. drymifolia | 45 | 7 | 37% | 2.5 | Bridge | (133) |
| Cyclosaplin (natural non-AMPs; cyclic peptides; plants; XXC, UCBB1aA) | RLGDGCTR | somatic seedlings, Santalum album L. | 8 | 1 | 25% | 4.13 | Unknown | (134) |
| Baceridin (natural AMPs; bacteriocins, Gram-positive bacteria, prokaryotes; XXC; XXD3, UCBB1ad) | WAIVLL | plant-associated Bacillus strain | 6 | 0 | 100% | -3.82 | Unknown | (135) |
| Frenatin 2.1S (natural AMPs; frogs, amphibians, animals; XXA; UCLL1c) | GLVGTLLGHIGKAILG | skin secretions, the Orinoco lime frog, Sphaenorhynchus lacteus, north central Guyana, South America | 16 | 3 | 50% | -1.7 | Unknown | (136) |
| Frenatin 2.2S (natural AMPs; frogs, amphibians, animals; XXA; UCLL1c; others: Frenatin 2.3S | GLVGTLLGHIGKAILS | skin secretions, the Orinoco lime frog, Sphaenorhynchus lacteus, north central Guyana, South America | 16 | 3 | 50% | -1.43 | Unknown | (136) |
| Gageostatin A (natural AMPs; Leu-rich; UCLL1c; nonribosomal peptide; lipopeptides; marine bacteria, prokaryotes; XXD; XXL; other forms: Gageostatin B; Gageostatin C) | ELLVDLL | Bacillus subtilis | 7 | -3 | 71% | -1.17 | Unknown | (137) |
| Brevinin-2R (natural AMPs; frogs, amphibians, animals; XXU; 1S=S, UCSS1a) | KLKNFAKGVAQSLLNKASCKLSGQC | skin, Marsh frog, formerly Rana ridibunda , new name Pelophylax ridibunda, Europe | 25 | 5 | 44% | 1.02 | Unknown | (138) |
| Crotalicidin (Ctn, Vipericidins, Lys-rich; natural AMPs; cathelicidins; Rattlesnake, reptiles, animals; derivatives: Ctn[15-34]; more AMPs? Lachesicidin; Lutzicidin ) | KRFKKFFKKVKKSVKKRLKKIFKKPMVIGVTIPF | venom gland, South American pit vipers, [Crotalus durissus terrificus](http://reptile-database.reptarium.cz/species?genus=Crotalus&species=durissus) | 34 | 15 | 41% | 1.58 | Helix | (139) |
| CecropinXJ (natural AMPs; insects, arthropods, invertebrates, animals) | RWKIFKKIEKMGRNIRDGIVKAGPAIEVLGSAKAIGK | larvae, Bombyx mori | 37 | 7 | 43% | 1.45 | Unknown | (140) |
| AaeAP1 (natural AMPs; scorpions, arachnids, Chelicerata, arthropods, invertebrates, animals; XXA, UCLL1c) | FLFSLIPSVIAGLVSAIRN | Venom, Androctonus aeneas, Africa | 19 | 2 | 63% | -0.86 | Unknown | (141) |
| AaeAP2 (natural AMPs; scorpions, arachnids, Chelicerata, arthropods, invertebrates, animals; XXA, UCLL1c) | FLFSLIPSAIAGLVSAIRN | Venom, Androctonus aeneas, Africa | 19 | 2 | 63% | -0.74 | Unknown | (141) |
| Stigmurin (natural AMPs; scorpions, arachnids, Chelicerata, arthropods, invertebrates, animals; UCLL1a) | FFSLIPSLVGGLISAFK | venom gland, Tityus stigmurus | 17 | 1 | 58% | -1.5 | Helix | (142) |
| Scolopendrasin VII (natural non-AMPs; myriapods, arthropods, invertebrates, animals; XXA; 1S=S; UCSS1a) | FCTCNVKGFNAKNKRGIIYP | centipede, Scolopendra subspinipes mutilans, Asia | 20 | 5 | 40% | 1.4 | Unknown | (143) |
| Vigno 5 (natural non-AMPs; cyclotides, plants; 3S=S, XXC; UCBB1ab; more AMPs? Vigno 1-4, 6-10) | GLPLCGETCVGGTCNTPGCSCGWPVCVRN | Viola ignobilis | 29 | 0 | 41% | 0.29 | Bridge | (144) |
| DC1 (natural non-AMPs; dissusa cyclotide 1, plants; 3S=S, XXC; UCBB1ab) | GAFLKCGESCVYLPCLTTVVGCSCQNSVCYRD | Hedyotis diffusa | 32 | 0 | 46% | 0.54 | Bridge | (145) |
| DC2 (natural non-AMPs; dissusa cyclotide 2, plants; 3S=S, XXC; UCBB1ab) | GAVPCGETCVYLPCITPDIGCSCQNKVCYRD | Hedyotis diffusa | 31 | -1 | 41% | 0.85 | Bridge | (145) |
| DC3 (dissusa cyclotide 3, natural non-AMPs; plants; 3S=S, XXC; UCBB1ab) | GTSCGETCVLLPCLSSVLGCTCQNKRCYKD | Hedyotis diffusa | 30 | 1 | 40% | 1.11 | Bridge | (145) |
| XLAsp-P1 (Asp-rich; natural AMPs; frog, amphibians, animals, UCLL1a) | DEDDD | skin, African clawed frog, Xenopus laevis, Africa | 5 | -5 | 0% | 8.33 | Unknown | (146) |
| RP9 (natural AMPs; Crocodile, reptiles, animals; UCLL1) | RGSALTHLP | leukocyte extract, Crocodylus siamensis | 9 | 2 | 33% | 1.44 | Unknown | (147) |
| Chaxapeptin (Phe-rich; natural AMPs; lactam; class 2 lasso peptide; class 1 microcin, bacteriocins; Gram-positive bacteria, prokaryotes; XXJ; UCSB1a) | GFGSKPLDSFGLNFF | Streptomyces leeuwenhoekii Strain C58; extremophile | 15 | 0 | 40% | 0.2 | Unknown | (148) |
| Sungsanpin (natural non-AMPs; lactam; class 2 lasso peptide; class 1 microcin, bacteriocins; Gram-positive bacteria, prokaryotes; XXJ; UCSB1a) | GFGSKPIDSFGLSWL | a Marine Streptomyces species | 15 | 0 | 40% | -0.09 | Unknown | (149) |
| Smp24 (natural AMPs; scorpions, arachnids, Chelicerata, arthropods, invertebrates, animals; UCLL1a) | IWSFLIKAATKLLPSLFGGGKKDS | venom, Scorpio maurus palmatus | 24 | 3 | 45% | -0.02 | Helix | (150) |
| Smp43 (natural AMPs; scorpions, arachnids, Chelicerata, arthropods, invertebrates, animals; UCLL1a) | GVWDWIKKTAGKIWNSEPVKALKSQALNAAKNFVAEKIGATPS | venom, Scorpio maurus palmatus | 43 | 4 | 44% | 0.92 | Helix | (150) |
| Saha-CATH3 (natural AMPs; cathelicidins; mammals, animals; UCLL1) | KRMGIFHLFWAGLRKLGNLIKNKIQQGIENFLG | Tasmanian devil, Sarcophilus harrisii | 33 | 6 | 45% | 0.91 | Unknown | (151) |
| Saha-CATH5 (natural AMPs; cathelicidins; mammals, animals; UCLL1) | KRIGLIRLIGKILRGLRRLG | Tasmanian devil, Sarcophilus harrisii | 20 | 7 | 45% | 1.88 | Unknown | (151) |
| Saha-CATH6 (Arg-rich; natural AMPs; cathelicidins; mammals, animals, UCLL1) | KRIRFFERIRDRLRDLGNRIKNRIRDFFS | Tasmanian devil, Sarcophilus harrisii | 29 | 7 | 34% | 5.26 | Unknown | - |
| mBD-2 (Murine beta-defensin 2, UCSS1a; natural AMPs; mouse, rodents, mammals, animals; 3S=S) | CHTNGGYCVRAICPPSARRPGSCFPEKNPCCKYM | Mus musculus | 34 | 5 | 35% | 1.81 | Bridge | (152) |
| Laterosporulin10 (LS10, natural AMPs; class-2d bacteriocin, defensin-like, Gram-positive bacteria, prokaryote; 3S=S; UCSS1a; BBMm) | ACVNQCPDAIDRFIVKDKGCHGVEKKYYKQVYVACMNGQHLYCRTEWGGPCQL | Brevibacillus sp. strain SKDU10 | 53 | 4 | 39% | 1.5 | Bridge | (153) |
| Bombinin-BO1 (natural AMPs; toad, amphibians, animals; XXA, UCSS1a) | GIGSAILSAGKSIIKGLAKGLAEHF | skin secretion, Oriental fire-bellied toad, Bombina orientalis, China, Asia | 25 | 5 | 48% | -0.44 | Unknown | (154) |
| Bombinin H-BO1 (Gly-rich; Leu-rich; natural AMPs; toad, amphibians, animals; XXA, UCSS1a) | IIGPVLGLVGKALGGLL | skin secretion, Oriental fire-bellied toad, Bombina orientalis, China, Asia | 17 | 2 | 58% | -2.55 | Unknown | (154) |
| Dermaseptin-PH (Dermaseptin PH; natural AMPs; frog, amphibians, animals; XXA; UCLL1c) | ALWKEVLKNAGKAALNEINNLV | orange-legged leaf frog, Pithecopus (Phyllomedusa) hypochondrialis, South America | 22 | 2 | 54% | 0.62 | Helix | (155) |
| TP (natural AMPs; bacteriocin, bacteria, prokaryotes; BBMm; BBN; UCLL1) | ASVVNKLTGGVAGLLK | caecum-derived strain TS | 16 | 2 | 50% | -0.6 | Unknown | (156) |
| Distinctin-Like-Peptide-PH (DLP-PH; natural AMPs; frog, amphibians, animals; UCLL1a ) | NLVSALIEGRKYLKNVLKKLNRLKEKNKAKNSKENN | Skin Secretion, Phyllomedusa hypochondrialis, South America | 36 | 8 | 30% | 2.96 | Unknown | (157) |
| Temporin-PE (Leu-rich; natural AMPs; edible frog, amphibians, animals; Leu-rich; XXA; UCLL1c ) | FLPIVAKLLSGLL | skin secretions, Pelophylax kl. esculentus, Europe | 13 | 2 | 69% | -2.33 | Helix | (158) |
| TAT-RasGAP317-326 (Arg-rich; synthetic20, UCLL1) | RRRQRRKKRGGGDTRLNTVWMW | artificial, designed | 22 | 8 | 22% | 5.58 | Unknown | (159) |
| Dermaseptin-PS3 (DPS3, XXA, UCLL1c; natural AMPs; frog, amphibians, animals) | ALWKDILKNAGKAALNEINQIVQ | skin secretion, Phyllomedusa sauvagii, USA, North America | 23 | 2 | 52% | 0.83 | Unknown | (160) |
| DRS-DU-1 (dermaseptin; natural AMPs; frog, amphibians, animals; XXA; UCLL1c) | ALWKSLLKNVGKAAGKAALNAVTDMVNQ | Callimedusa (Phyllomedusa) duellmani, Peru, South America | 28 | 4 | 53% | 0.46 | Unknown | (161) |
| Dermaseptin-PS1 (natural AMPs; frog, amphibians, animals; XXA; UCLL1c) | ALWKTMLKKLGTVALHAGKAALGAVADTISQ | skin, the waxy monkey tree frog, Phyllomedusa sauvagei, South America | 31 | 5 | 54% | -0.17 | Helix | (162) |
| Ranatuerin-2PLx (R2PLx, natural AMPs; frog, amphibians, animals; XXU; 1S=S, UCSS1a) | GIMDTVKNAAKNLAGQLLDKLKCSITAC | skin secretions, the pickerel frog, Rana palustris, North America | 28 | 2 | 50% | 0.69 | Helix | (163) |
| Mastoparan-C (Mastoparan C, MP-C; MPC, Leu-rich; natural AMPs; insects, arthropods, invertebrates, animals. XXA; UCLL1c) | LNLKALLAVAKKIL | venom, the European Hornet, Vespa crabro | 14 | 4 | 71% | -1.12 | Helix | (164) |
| VLL-28 (natural AMPs; archaeocins, archaea, prokaryote; UCLL1) | VLLVTLTRLHQRGVIYRKWRHFSGRKYR | Sulfolobus islandicus | 28 | 10 | 35% | 2.87 | Helix | (165) |
| Phylloseptin-PHa (PSPHa, XXA; UCLL1c; Ala-rich; natural AMPs; frog, amphibians, animals) | FLSLIPAAISAVSALANHF | skin secretions, Orange-legged Leaf Frog, Pithecopus hypochondrialis, South America | 19 | 2 | 68% | -1.16 | Helix | (166) |
| Ss-arasin (UCSS1a, 2S=S, natural AMPs; Crustaceans, arthropods, invertebrates, animals) | SPRVRRRYGRPFGGRPFVGGQFGGRPGCVCIRSPCPCANYG | the Indian mud crab, Scylla serrata | 41 | 8 | 29% | 2.37 | Bridge | (167) |
| LFB (L. fujianensis Brevinvin; natural AMPs; frog, amphibians, animals; XXU; 1S=S, UCSS1a) | GLFSVVKGVLKGVGKNVSGSLLDQLKCKISGGC | the Fujian Large Headed Frog, Limnonectes fujianensi, China, Asia | 33 | 4 | 42% | 0.01 | Helix | (168) |
| Dermaseptin-PT9 (DPT9, UCLL1a; natural AMPs; frog, amphibians, animals) | GLWSKIKDAAKTAGKAALGFVNEMV | skin secretion, Phyllomedusa tarsius, Purchased in Peru, South America | 25 | 2 | 52% | 0.31 | Helix | (169) |
| Turgencin A (natural AMPs; sea squirt, tunicate, invertebrates, marine animals; 3S=S, UCSS1; XXA; XXO) | GPKTKAACKMACKLATCGKKPGGWKCKLCELGCDAV | Synoicum turgens | 36 | 7 | 47% | 0.56 | Unknown | (170) |
| Turgencin B (natural AMPs; sea squirt, tunicate, invertebrates, marine animals; 3S=S, UCSS1; XXA; XXO) | GIKEMLCNMACAQTVCKKSGGPLCDTCQAACKALG | Synoicum turgens | 35 | 3 | 51% | 0.43 | Helix | (170) |
| Figainin 2 (natural AMPs; frog, amphibians, animals; UCLL1; derivative: F2-12; stapled peptides) | FLGAILKIGHALAKTVLPMVTNAFKPKQ | skin secretion, the Chaco tree frog, Boana raniceps, South America | 28 | 5 | 53% | -0.38 | Helix | (171) |
| Phylloseptin-PBa1 (natural AMPs; frog, amphibians, animals; UCLL1c; XXA) | FLSLIPHIASGIASLVKNF | Burmeister's leaf frog. Phyllomedusa burmeisteri , Brazil, South America | 19 | 3 | 57% | -0.89 | Unknown | (172) |
| Phylloseptin-PBa2 (natural AMPs; frog, amphibians, animals; UCLL1c; XXA; inactive: Phylloseptin-PBa2 MIC 200 uM or greater) | FLSLLPHIASGIASLVSKF | Burmeister's leaf frog. Phyllomedusa burmeisteri , Brazil, South America | 19 | 3 | 57% | -1.06 | Unknown | (172) |
| Kassiniatuerin-3 (Ile-rich; natural AMPs; frog, amphibians, animals; UCLL1; XXA) | FIQHLIPLIPHAIQGIKDIF | Kassina senegalensis, Africa | 20 | 3 | 55% | -0.67 | Helix | (173) |
| Dermaseptin-PD-1 (DRS-PD-1; Ala-rich; natural AMPs; frog, amphibians, animals; UCLL1a) | GMWSKIKETAMAAAKEAAKAAGKTISDMIKQ | Skin Secretion, Pachymedusa dacnicolor, Mexico, North America | 31 | 3 | 48% | 1.05 | Unknown | (174) |
| Dermaseptin-PD-2 (DRS-PD-2; Ala-rich; natural AMPs; frog, amphibians, animals; UCLL1a) | GMWSKIKNAGKAAAKAAAKAAGKAALDAVSEAI | Skin Secretion, Pachymedusa dacnicolor, Mexico, North America | 33 | 4 | 57% | 0.37 | Rich | (174) |
| Cecropin A (cecropin A1; CecA1; cecropin A2; natural AMPs; insects, arthropods, invertebrates, animals; UCLL1; Variant: BmCecA (B. mori cecropin A) | RWKLFKKIEKVGRNVRDGLIKAGPAIAVIGQAKSL | silkworm, Bombyx mori | 35 | 7 | 45% | 1.32 | Unknown | (175) |
| Phylloseptin-PTa (XXA; UCLL1c; natural AMPs; frog, amphibians, animals) | FLSLIPKIAGGIAALAKHL | skin secretions, the Brown-bellied Leaf Frog, Phyllomedusa tarsius, South America | 19 | 4 | 63% | -1.44 | Helix | (166) |
| Figainin 1 (UCLL1c; Leu-rich; natural AMPs; frog, amphibians, animals, XXA) | FIGTLIPLALGALTKLFK | skin secretions, Chaco tree frog, Boana raniceps, South America | 18 | 3 | 61% | -1.64 | Helix | (176) |
| Catfish PACAP38 (XXA, neuropeptide, natural AMPs; fish, animals) | HSDGIFTDSYSRYRKQMAVKKYLAAVLGRRYRQRFRNK | Clarias gariepinus, North Africa | 38 | 11 | 29% | 3.71 | Unknown | (177) |
| Esculentin-2 HYba1 (natural AMPs; frog, amphibians, animals; XXA, XXU, 1S=S, UCSS1a) | SIFSLFKMGAKALGKTLLKQAGKAGAEYAACKATNQC | skin secretion, Hydrophylax bahuvistara, India, Asia | 37 | 6 | 49% | 0.43 | Unknown | (178) |
| Esculentin-2 HYba2 (natural AMPs; frog, amphibians, animals; XXA; XXU, 1S=S, UCSS1a) | SILSLFKMGAKALGKTLIKQAGKAGAEYVACKATNQC | skin secretion, Hydrophylax bahuvistara, India, Asia | 37 | 6 | 49% | 0.32 | Unknown | (178) |
| SSTP1 (Temporin1IDau1, Leu-rich 33%; natural non-AMPs; frog, amphibians, animals, UCLL1) | FLPLLISALTSLFPKLGK | skin secretion, the golden frog, the Trivandrum frog, the common wood frog, or the small wood frog, Indosylvirana aurantiaca, India, Asia | 18 | 2 | 56% | -1.26 | Unknown | (179) |
| t-DPH1 (t-dermaseptin-PH1, Ala-rich 33%; natural AMPs; frog, amphibians, animals, UCLL1; BBMm) | GLWSKIKNVAAAAGKAALGAL | skin secretion, the northern orange-legged leaf frog, tiger-legged monkey frog, Phyllomedusa hypochondrialis, South America | 21 | 4 | 62% | -0.7 | Helix | (180) |
| Brevinin-2KP (natural AMPs; frog, amphibians, animals, 1S=S; UCSS1; BBMm) | GVITDALKGAAKTVAAELLKKAHCKLTNSC | Skin, the banded bullfrog, common Asian frog, Kaloula pulchra, Asia | 30 | 4 | 50% | 0.59 | Helix | (181) |
| Nigrocin-PN (UCSS1a; likely natural AMPs; Gly-rich; frog, amphibians, animals; derivatives: Nigrocin-M1; Nigrocin-M2: inactive) | GLLGKILGAGKKVLCGVSGLC | Pelophylax nigromaculatus, Asia | 21 | 3 | 52% | -1.31 | Helix | (182) |
| [D4K]OCN-3N (synthetic20, XXA; UCLL1c) | GIFKVLKNLAKGVITSLAS | derivatives of ocellatin-3N | 19 | 4 | 53% | -0.44 | Unknown | (183) |
| Lt-MAP2 (Lys-rich; Leu-rich; synthetic20, UCLL1a) | LIKKLKEYLKKLI | derivatives of Latarcin-3a | 13 | 4 | 46% | 0.39 | Unknown | (184) |
| [A18K]OCN-3N (synthetic20, XXA; UCLL1c) | GIFDVLKNLAKGVITSLKS | derivatives of ocellatin-3N | 19 | 3 | 47% | 0.1 | Unknown | (183) |
| [D4K,A18K]OCN-3N (synthetic20, XXA; UCLL1c) | GIFKVLKNLAKGVITSLKS | derivatives of ocellatin-3N | 19 | 5 | 47% | -0.06 | Unknown | (183) |
| Emericellipsin A (EmiA , Lipopeptaibol, natural AMPs, nonribosomal peptide, fungii; XXL, XXM, XXK) | PQAAIVASG | Emericellopsis alkaline VKPM F1428, Alkalophile, extremophile | 9 | 0 | 56% | -0.7 | Unknown | (185) |
| Raniseptin-6 (Rsp-6, natural AMPs; frogs, amphibians, animals; UCLL1) | ALLDKLKSLGKVVGKVALGVVQNYLNPRQ | skin secretion, Chaco tree frog, Boana raniceps, South America | 29 | 4 | 45% | 0.6 | Helix | (186) |
| Raniseptin-3 (Rsp-3, natural AMPs; frogs, amphibians, animals; UCLL1) | AWLDKLKSIGKVVGKVAIGVAKNLLNPQ | skin secretion, Chaco tree frog, Boana raniceps, South America | 28 | 4 | 50% | 0.08 | Helix | (186) |
| mCRAMP (mouse cathelin-related antimicrobial peptide; CRAMP-34; natural AMPs; cathelicidin, rodents, mammals, animals, UCLL1) | GLLRKGGEKIGEKLKKIGQKIKNFFQKLVPQPEQ | bone marrow, saliva, Mice, Mus musculus | 34 | 6 | 29% | 1.74 | Unknown | (187) |
| CRAMP-18 E2K (Lys-rich; synthetic, mutant of CRAMP-18, UCLL1) | GKKLKKIGQKIKNFFQKL | Engineered | 18 | 7 | 33% | 1.61 | Helix | (188) |
| Brevinin-1-AW (B1AW, natural AMPs; frog, amphibians, animals; 1S=S, XXU, UCSS1a; BBMm) | FLPLLAGLAANFLPQIICKIARKC | skin secretion, the Wuyi torrent frog, Amolops wuyiensis, China, Asia | 24 | 3 | 67% | -0.74 | Helix | (189) |
| B1AW-K (Brevinin-1-AW Q15K mutant; synthetic, 1S=S, XXU, UCSS1a) | FLPLLAGLAANFLPKIICKIARKC | amino acid substitution, animal-derived, natural derivative | 24 | 4 | 67% | -0.74 | Helix | (189) |
| Figainin 2BN (Leu-rich; natural AMPs; frog, amphibians, animals, UCLL1) | FLGVALKLGKVLGKALLPLASSLLHSQ | norepinephrine-stimulated skin secretion, the Giant Gladiator Treefrog, the Rusty Treefrog, Boana boans, Trinidad, South America | 27 | 4 | 56% | -0.98 | Helix | (190) |
| Picturin 1BN (natural AMPs; frog, amphibians, animals, UCLL1) | GIFKDTLKKVVAAVLTTVADNIHPK | norepinephrine-stimulated skin secretion, the Giant Gladiator Treefrog, the Rusty Treefrog, Boana boans, Trinidad, South America | 25 | 3 | 48% | 0.53 | Helix | (190) |
| Picturin 2BN (natural AMPs; frog, amphibians, animals, UCLL1) | GLMDMLKKVGKVALTVAKSALLP | norepinephrine-stimulated skin secretion, the Giant Gladiator Treefrog, the Rusty Treefrog, Boana boans, Trinidad, South America | 23 | 3 | 57% | -0.51 | Helix | (190) |
| Dermaseptin-SS1 (SS1, XXA, UCLL1c; BBL; BBMm; natural AMPs; frog, amphibians, animals) | ALWKSILKNAGKAALNEINQIV | Skin Secretion, Brown-belly leaf frog, Phyllomedusa Tarsius , South America | 23 | 3 | 52% | 0.37 | Helix | (191) |
| MPC-A5K,A8K (Mastoparan C A5K,A8K, Synthetic, Lys-rich; Leu-rich; XXA, UCLL1c) | LNLKKLLKVAKKIL | Amino acid substitution | 14 | 6 | 57% | -0.07 | Unknown | (192) |
| Ranatuerin-2-AW D4K, D19K, K20L (R2AW analog, Synthetic, XXA, UCLL1c) | GFMKTAKNVAKNVAATLLKLLK | sequence truncation, Amino acid substitution, animal-derived, natural derivative | 22 | 6 | 55% | 0.22 | Helix | (193) |
| Ranatuerin-2-AW A6W, A10W (R2AW analog 2, Synthetic, XXA, UCLL1c) | GFMKTWKNVWKNVAATLLKLLK | sequence truncation, Amino acid substitution, animal-derived, natural derivative | 22 | 6 | 55% | 0.17 | Helix | (193) |
| Raniseptin PL (XXA; UCLL1c; natural AMPs; frog, amphibians, animals) | GVFDTVKKIGKAVGKFALGVAKNYLNS | skin secretions, the banana tree dwelling frog, Boana platanera (Hylidae; Hylinae), Trinidad, South America | 27 | 5 | 44% | 0.36 | Unknown | (194) |
| Figainin 2PL (XXA; UCLL1c; natural AMPs; frog, amphibians, animals) | FLGTVLKLGKAIAKTVVPMLTNAMQPKQ | skin secretions, the banana tree dwelling frog, Boana platanera (Hylidae; Hylinae), Trinidad, South America | 28 | 5 | 50% | -0.14 | Unknown | (194) |
| Hylin PL (XXA; UCLL1c; natural AMPs; frog, amphibians, animals) | FLGLIPALAGAIGNLIK | skin secretions, the banana tree dwelling frog, Boana platanera (Hylidae; Hylinae), Trinidad, South America | 17 | 2 | 65% | -1.96 | Unknown | (194) |
| Brevinin-1BW (natural AMPs; frog, amphibians, animals; XXU; 1S=S, UCSS1a; BBL) | FLPLLAGLAASFLPTIFCKISRKC | skin, Pelophylax nigromaculatus, East Asia | 24 | 3 | 63% | -0.7 | Unknown | (195) |
| StigA6 (S7K, G10K Stigmurin analog, synthetic, XXA, UCLL1c) | FFSLIPKLVKGLISAFK | amino acid substitution, animal-derived, natural derivative | 17 | 4 | 59% | -0.99 | Helix | (196) |
| StigA16 (S3K, S7K, G10K Stigmurin analog, synthetic, XXA, UCLL1c) | FFKLIPKLVKGLISAF | amino acid substitution, amino acid truncation, animal-derived, natural derivative | 16 | 4 | 63% | -1.26 | Helix | (196) |
| Dermaseptin-PS4 (Der-PS4, XXA, UCLL1c; natural AMPs; frog, amphibians, animals) | ALWKTLLKHVGKAAGKAALNAVTDMVNQ | skin secretion, Painted-belly leaf frog, Phyllomedusa sauvagii, South America | 28 | 5 | 54% | 0.36 | Helix | (197) |
| AP-64 (AMP with 64 aa, C5orf46, natural AMPs, human, primates, mammals, animals; UCLL1) | DDKPDKPDDKPDDSGKDPKPDFPKFLSLLGTEIIENAVEFILRSMSRSTGFMEFDDNEGKHSSK | Homo sapiens | 64 | -5 | 25% | 2.92 | Unknown | (198) |
| AaeAP1a (AaeAP1 analog, Lys-rich, synthetic, XXA, UCLL1c) | FLFKLIPKVIKGLVKAIRK | amino acid substitution, scorpions, animal-derived, natural derivative | 19 | 7 | 58% | -0.19 | - | (141) |
| AaeAP2a (AaeAP2 analog, Lys-rich, synthetic, XXA, UCLL1c) | FLFKLIPKAIKGLVKAIRK | amino acid substitution, scorpions, animal-derived, natural derivative | 19 | 7 | 58% | -0.07 | - | (141) |

1. Mor A, Nicolas P. Isolation and structure of novel defensive peptides from frog skin. European Journal of Biochemistry. 1994;219(1‐2):145-54.

2. Rozek T, Wegener KL, Bowie JH, Olver IN, Carver JA, Wallace JC, et al. The antibiotic and anticancer active aurein peptides from the Australian Bell Frogs Litoria aurea and Litoria raniformis: The solution structure of aurein 1.2. European Journal of Biochemistry. 2000;267(17):5330-41.

3. WU L, WU Z, Lin D, Fang F, Lin Q, Xie L. Characterization and amino acid sequence of y3, an antiviral protein from mushroom Coprinus comatus. Chinese Journal of Biochemistry and Molecular Biology. 2008:597-603.

4. Chernysh S, Kim S, Bekker G, Pleskach V, Filatova N, Anikin V, et al. Antiviral and antitumor peptides from insects. Proceedings of the National Academy of Sciences. 2002;99(20):12628-32.

5. Bellamy W, Takase M, Wakabayashi H, Kawase K, Tomita M. Antibacterial spectrum of lactoferricin B, a potent bactericidal peptide derived from the N‐terminal region of bovine lactoferrin. Journal of Applied Bacteriology. 1992;73(6):472-9.

6. Oh H, Hedberg M, Wade D, Edlund C. Activities of synthetic hybrid peptides against anaerobic bacteria: aspects of methodology and stability. Antimicrobial agents and chemotherapy. 2000;44(1):68-72.

7. Nissen-Meyer J, Larsen AG, Sletten K, Daeschel M, Nes IF. Purification and characterization of plantaricin A, a Lactobacillus plantarum bacteriocin whose activity depends on the action of two peptides. Microbiology. 1993;139(9):1973-8.

8. Miele R, Borro M, Fiocco D, Barra D, Simmaco M. Sequence of a gene from Bombina orientalis coding for the antimicrobial peptide BLP-7. Peptides. 2000;21(11):1681-6.

9. Lai R, Zheng Y-T, Shen J-H, Liu G-J, Liu H, Lee W-H, et al. Antimicrobial peptides from skin secretions of Chinese red belly toad Bombina maxima. Peptides. 2002;23(3):427-35.

10. Park JM, Jung J-E, Lee BJ. Antimicrobial peptides from the skin of a Korean frog, Rana rugosa. Biochemical and biophysical research communications. 1994;205(1):948-54.

11. Simmaco M, Mignogna G, Canofeni S, Miele R, Mangoni ML, Barra D. Temporins, antimicrobial peptides from the European red frog Rana temporaria. European Journal of Biochemistry. 1996;242(3):788-92.

12. QU Xm, STEINER H, Engström Å, Bennich H, Boman HG. Insect immunity: isolation and structure of cecropins B and D from pupae of the Chinese oak silk moth, Antheraea pernyi. European journal of biochemistry. 1982;127(1):219-24.

13. Rosetto M, Manetti AG, Marchini D, Dallai R, Telford JL, Baldari CT. Sequences of two cDNA clones from the medfly Ceratitis capitata encoding antibacterial peptides of the cecropin family. Gene. 1993;134(2):241-3.

14. Steiner H, Hultmark D, Engström Å, Bennich H, Boman HG. Sequence and specificity of two antibacterial proteins involved in insect immunity. Nature. 1981;292(5820):246-8.

15. Lu J, Chen Z-w. Isolation, characterization and anti-cancer activity of SK84, a novel glycine-rich antimicrobial peptide from Drosophila virilis. Peptides. 2010;31(1):44-50.

16. Zasloff M. Magainins, a class of antimicrobial peptides from Xenopus skin: isolation, characterization of two active forms, and partial cDNA sequence of a precursor. Proceedings of the National Academy of Sciences. 1987;84(15):5449-53.

17. Fennell J, Shipman W, Cole L. Antibacterial action of a bee venom fraction (melittin) against a penicillin-resistant staphylococcus and other microorganisms. USNRDL-TR-67-101. Research and Development Technical report United States Naval Radiological Defense Laboratory, San Francisco. 1967:1-13.

18. Selsted ME, Novotny MJ, Morris WL, Tang Y-Q, Smith W, Cullor JS. Indolicidin, a novel bactericidal tridecapeptide amide from neutrophils. Journal of Biological Chemistry. 1992;267(7):4292-5.

19. Lawyer C, Pai S, Watabe M, Borgia P, Mashimo T, Eagleton L, et al. Antimicrobial activity of a 13 amino acid tryptophan‐rich peptide derived from a putative porcine precursor protein of a novel family of antibacterial peptides. FEBS letters. 1996;390(1):95-8.

20. Charpentier S, Amiche M, Mester J, Vouille V, Le Caer J-P, Nicolas P, et al. Structure, synthesis, and molecular cloning of dermaseptins B, a family of skin peptide antibiotics. Journal of Biological Chemistry. 1998;273(24):14690-7.

21. Cole AM, Weis P, Diamond G. Isolation and characterization of pleurocidin, an antimicrobial peptide in the skin secretions of winter flounder. Journal of Biological Chemistry. 1997;272(18):12008-13.

22. Selsted M, Harwig S, Ganz T, Schilling JW, Lehrer R. Primary structures of three human neutrophil defensins. The Journal of clinical investigation. 1985;76(4):1436-9.

23. Silva PI, Daffre S, Bulet P. Isolation and characterization of gomesin, an 18-residue cysteine-rich defense peptide from the spider Acanthoscurria gomesiana hemocytes with sequence similarities to horseshoe crab antimicrobial peptides of the tachyplesin family. Journal of Biological Chemistry. 2000;275(43):33464-70.

24. Park CH, Valore EV, Waring AJ, Ganz T. Hepcidin, a urinary antimicrobial peptide synthesized in the liver. Journal of biological chemistry. 2001;276(11):7806-10.

25. Park NG, Yamato Y, Lee S, Sugihara G. Interaction of mastoparan‐B from venom of a hornet in Taiwan with phospholipid bilayers and its antimicrobial activity. Biopolymers: Original Research on Biomolecules. 1995;36(6):793-801.

26. Hirai Y, YASUHARA T, YOSHIDA H, NAKAJIMA T, FUJINO M, KITADA C. A new mast cell degranulating peptide" mastoparan" in the venom of Vespula lewisii. Chemical and Pharmaceutical Bulletin. 1979;27(8):1942-4.

27. Mulders JW, Boerrigter IJ, Rollema HS, Siezen RJ, De Vos WM. Identification and characterization of the lantibiotic nisin Z, a natural nisin variant. European Journal of Biochemistry. 1991;201(3):581-4.

28. Rogers L. The inhibiting effect of Streptococcus lactis on Lactobacillus bulgaricus. Journal of bacteriology. 1928;16(5):321-5.

29. Nakamura T, Furunaka H, Miyata T, Tokunaga F, Muta T, Iwanaga S, et al. Tachyplesin, a class of antimicrobial peptide from the hemocytes of the horseshoe crab (Tachypleus tridentatus). Isolation and chemical structure. Journal of Biological Chemistry. 1988;263(32):16709-13.

30. Vernon LP, Evett GE, Zeikus RD, Gray WR. A toxic thionin from Pyrularia pubera: purification, properties, and amino acid sequence. Archives of Biochemistry and Biophysics. 1985;238(1):18-29.

31. Wong H, Bowie JH, Carver JA. The solution structure and activity of caerin 1.1, an antimicrobial peptide from the Australian green tree frog, Litoria splendida. European journal of biochemistry. 1997;247(2):545-57.

32. STONE DM, Waugh R, Bowie J, Wallace J, Tyler M. Peptides from Australian frogs. The structures of the caerins from Litoria caerulea. Journal of chemical research Synopses (Print). 1993(4).

33. Steinborner ST, Waugh RJ, Bowie JH, Wallace JC, Tyler MJ, Ramsay SL. New caerin antibacterial peptides from the skin glands of the Australian tree frog Litoria xanthomera. Journal Of Peptide Science: An Official Publication Of The European Peptide Society. 1997;3(3):181-5.

34. Steinborner ST, Currie GJ, Bowie JH, Wallace JC, Tyler MJ. New antibiotic caerin 1 peptides from the skin secretion of the Australian tree frog Litoria chloris. Comparison of the activities of the caerin 1 peptides from the genus Litoria. The Journal of peptide research. 1998;51(2):121-6.

35. Rozek T, Waugh RJ, Steinborner ST, Bowie JH, Tyler MJ, Wallace JC. The maculatin peptides from the skin glands of the tree frog Litoria genimaculata: a comparison of the structures and antibacterial activities of maculatin 1.1 and caerin 1.1. Journal of peptide science: an official publication of the European Peptide Society. 1998;4(2):111-5.

36. Gallo RL, Kim KJ, Bernfield M, Kozak CA, Zanetti M, Merluzzi L, et al. Identification of CRAMP, a cathelin-related antimicrobial peptide expressed in the embryonic and adult mouse. Journal of Biological Chemistry. 1997;272(20):13088-93.

37. Harder J, Bartels J, Christophers E, Schröder J-M. Isolation and characterization of human μ-Defensin-3, a novel human inducible peptide antibiotic. Journal of Biological Chemistry. 2001;276(8):5707-13.

38. Sai KP, Jagannadham MV, Vairamani M, Raju NP, Devi AS, Nagaraj R, et al. Tigerinins: Novel antimicrobial peptides from the Indian frogRana tigerina. Journal of Biological Chemistry. 2001;276(4):2701-7.

39. Park CB, Kim MS, Kim SC. A novel antimicrobial peptide frombufo bufo gargarizans. Biochemical and biophysical research communications. 1996;218(1):408-13.

40. Gudmundsson GH, Agerberth B, Odeberg J, Bergman T, Olsson B, Salcedo R. The human gene FALL39 and processing of the cathelin precursor to the antibacterial peptide LL‐37 in granulocytes. European journal of biochemistry. 1996;238(2):325-32.

41. Iijima N, Tanimoto N, Emoto Y, Morita Y, Uematsu K, Murakami T, et al. Purification and characterization of three isoforms of chrysophsin, a novel antimicrobial peptide in the gills of the red sea bream, Chrysophrys major. European Journal of Biochemistry. 2003;270(4):675-86.

42. Wabnitz PA, Bowie JH, Tyler MJ, Wallace JC, Smith BP. Differences in the skin peptides of the male and female Australian tree frog Litoria splendida: the discovery of the aquatic male sex pheromone splendipherin, together with Phe8 caerulein and a new antibiotic peptide caerin 1.10. European Journal of Biochemistry. 2000;267(1):269-75.

43. Wegener KL, Wabnitz PA, Carver JA, Bowie JH, Chia BC, Wallace JC, et al. Host defence peptides from the skin glands of the Australian Blue Mountains tree‐frog Litoria citropa: Solution structure of the antibacterial peptide citropin 1.1. European journal of biochemistry. 1999;265(2):627-37.

44. Skerlavaj B, Gennaro R, Bagella L, Merluzzi L, Risso A, Zanetti M. Biological characterization of two novel cathelicidin-derived peptides and identification of structural requirements for their antimicrobial and cell lytic activities. Journal of Biological Chemistry. 1996;271(45):28375-81.

45. Destoumieux D, Munoz M, Bulet P, Bachère* E. Penaeidins, a family of antimicrobial peptides from penaeid shrimp (Crustacea, Decapoda). Cellular and Molecular Life Sciences CMLS. 2000;57:1260-71.

46. Agerberth B, LEE JY, Bergman T, CARLQUIST M, BOMAN HG, MUTT V, et al. Amino acid sequence of PR‐39: isolation from pig intestine of a new member of the family of proline‐arginine‐rich antibacterial peptides. European journal of biochemistry. 1991;202(3):849-54.

47. Ali MF, Soto A, Knoop FC, Conlon JM. Antimicrobial peptides isolated from skin secretions of the diploid frog, Xenopus tropicalis (Pipidae). Biochimica et biophysica acta (BBA)-protein structure and molecular enzymology. 2001;1550(1):81-9.

48. Andersson M, Gunne H, Agerberth B, Boman A, Bergman T, Sillard R, et al. NK‐lysin, a novel effector peptide of cytotoxic T and NK cells. Structure and cDNA cloning of the porcine form, induction by interleukin 2, antibacterial and antitumour activity. The EMBO journal. 1995;14(8):1615-25.

49. Bensch KW, Raida M, Mägert H-J, Schulz-Knappe P, Forssmann W-G. hBD‐1: a novel β‐defensin from human plasma. FEBS letters. 1995;368(2):331-5.

50. Silphaduang U, Noga EJ. Peptide antibiotics in mast cells of fish. Nature. 2001;414(6861):268-9.

51. Kuhn-Nentwig L, Muller J, Schaller J, Walz A, Dathe M, Nentwig W. Cupiennin 1, a new family of highly basic antimicrobial peptides in the venom of the spider Cupiennius salei (Ctenidae). Journal of Biological Chemistry. 2002;277(13):11208-16.

52. Lai R, Liu H, Lee WH, Zhang Y. An anionic antimicrobial peptide from toad Bombina maxima. Biochemical and biophysical research communications. 2002;295(4):796-9.

53. Dubos RJ. Studies on a bactericidal agent extracted from a soil bacillus: I. Preparation of the agent. Its activity in vitro. The Journal of experimental medicine. 1939;70(1):1.

54. Mai JC, Mi Z, Kim S-H, Ng B, Robbins PD. A proapoptotic peptide for the treatment of solid tumors. Cancer research. 2001;61(21):7709-12.

55. Clark DP, Durell S, Maloy WL, Zasloff M. Ranalexin. A novel antimicrobial peptide from bullfrog (Rana catesbeiana) skin, structurally related to the bacterial antibiotic, polymyxin. Journal of Biological Chemistry. 1994;269(14):10849-55.

56. Wong JH, Ng TB. Lunatusin, a trypsin-stable antimicrobial peptide from lima beans (Phaseolus lunatus L.). Peptides. 2005;26(11):2086-92.

57. King JD, Al-Ghaferi N, Abraham B, Sonnevend A, Leprince J, Nielsen PF, et al. Pentadactylin: an antimicrobial peptide from the skin secretions of the South American bullfrog Leptodactylus pentadactylus. Comparative Biochemistry and Physiology Part C: Toxicology & Pharmacology. 2005;141(4):393-7.

58. Souza BM, Mendes MA, Santos LD, Marques MR, César LM, Almeida RN, et al. Structural and functional characterization of two novel peptide toxins isolated from the venom of the social wasp Polybia paulista. Peptides. 2005;26(11):2157-64.

59. Wong JH, Ng TB. Sesquin, a potent defensin-like antimicrobial peptide from ground beans with inhibitory activities toward tumor cells and HIV-1 reverse transcriptase. Peptides. 2005;26(7):1120-6.

60. Kim SS, Shim MS, Chung J, Lim D-Y, Lee BJ. Purification and characterization of antimicrobial peptides from the skin secretion of Rana dybowskii. Peptides. 2007;28(8):1532-9.

61. Conlon JM, Kolodziejek J, Nowotny N, Leprince J, Vaudry H, Coquet L, et al. Cytolytic peptides belonging to the brevinin-1 and brevinin-2 families isolated from the skin of the Japanese brown frog, Rana dybowskii. Toxicon. 2007;50(6):746-56.

62. Shang D, Yu F, Li J, Zheng J, Zhang L, Li Y. Molecular cloning of cDNAs encoding antimicrobial peptide precursors from the skin of the Chinese brown frog, Rana chensinensis. Zoological science. 2009;26(3):220-6.

63. Henderson JT, Chopko AL, Van Wassenaar PD. Purification and primary structure of pediocin PA-1 produced by Pediococcus acidilactici PAC-1.0. Archives of biochemistry and biophysics. 1992;295(1):5-12.

64. Apponyi MA, Pukala TL, Brinkworth CS, Maselli VM, Bowie JH, Tyler MJ, et al. Host-defence peptides of Australian anurans: structure, mechanism of action and evolutionary significance. Peptides. 2004;25(6):1035-54.

65. Shai Y, Fox J, Caratsch C, Shih Y-L, Edwards C, Lazarovici P. Sequencing and synthesis of pardaxin, a polypeptide from the Red Sea Moses sole with ionophore activity. FEBS letters. 1988;242(1):161-6.

66. Li X, Li Y, Han H, Miller DW, Wang G. Solution structures of human LL-37 fragments and NMR-based identification of a minimal membrane-targeting antimicrobial and anticancer region. Journal of the American Chemical Society. 2006;128(17):5776-85.

67. Konno K, Rangel M, Oliveira JS, dos Santos Cabrera MP, Fontana R, Hirata IY, et al. Decoralin, a novel linear cationic α-helical peptide from the venom of the solitary eumenine wasp Oreumenes decoratus. Peptides. 2007;28(12):2320-7.

68. Patrzykat A, Gallant JW, Seo J-K, Pytyck J, Douglas SE. Novel antimicrobial peptides derived from flatfish genes. Antimicrobial Agents and Chemotherapy. 2003;47(8):2464-70.

69. Vorland LH, Ulvatne H, Andersen J, Haukland HH, Rekdal Ø, Svendsen JS, et al. Lactoferricin of bovine origin is more active than lactoferricins of human, murine and caprine origin. Scandinavian journal of infectious diseases. 1998;30(5):513-7.

70. Mignogna G, Simmaco M, Kreil G, Barra D. Antibacterial and haemolytic peptides containing D‐alloisoleucine from the skin of Bombina variegata. The EMBO journal. 1993;12(12):4829-32.

71. Huang P-H, Chen J-Y, Kuo C-M. Three different hepcidins from tilapia, Oreochromis mossambicus: analysis of their expressions and biological functions. Molecular immunology. 2007;44(8):1922-34.

72. Kim JB, Conlon JM, Iwamuro S, Knoop F. Antimicrobial peptides from the skin of the Japanese mountain brown frog, Rana ornativentris. The Journal of Peptide Research. 2001;58(5):349-56.

73. Lu Y, Li J, Yu H, Xu X, Liang J, Tian Y, et al. Two families of antimicrobial peptides with multiple functions from skin of rufous-spotted torrent frog, Amolops loloensis. Peptides. 2006;27(12):3085-91.

74. Conlon JM, Coquet L, Leprince J, Jouenne T, Vaudry H, Kolodziejek J, et al. Peptidomic analysis of skin secretions from Rana heckscheri and Rana okaloosae provides insight into phylogenetic relationships among frogs of the Aquarana species group. Regulatory peptides. 2007;138(2-3):87-93.

75. Pál T, Abraham B, Sonnevend Á, Jumaa P, Conlon JM. Brevinin-1BYa: a naturally occurring peptide from frog skin with broad-spectrum antibacterial and antifungal properties. International journal of antimicrobial agents. 2006;27(6):525-9.

76. Jacob L, Zasloff M, editors. Potential therapeutic applications of magainins and other antimicrobial agents of animal origin. Ciba Foundation Symposium 186‐Antimicrobial Peptides: Antimicrobial Peptides: Ciba Foundation Symposium 186; 2007: Wiley Online Library.

77. Conlon JM, Woodhams DC, Raza H, Coquet L, Leprince J, Jouenne T, et al. Peptides with differential cytolytic activity from skin secretions of the lemur leaf frog Hylomantis lemur (Hylidae: Phyllomedusinae). Toxicon. 2007;50(4):498-506.

78. Lay FT, Schirra HJ, Scanlon MJ, Anderson MA, Craik DJ. The three-dimensional solution structure of NaD1, a new floral defensin from Nicotiana alata and its application to a homology model of the crop defense protein alfAFP. Journal of Molecular Biology. 2003;325(1):175-88.

79. Milligan SB, Gasser CS. Nature and regulation of pistil-expressed genes in tomato. Plant molecular biology. 1995;28:691-711.

80. Kozlov SA, Vassilevski AA, Feofanov AV, Surovoy AY, Karpunin DV, Grishin EV. Latarcins, antimicrobial and cytolytic peptides from the venom of the spider Lachesana tarabaevi (Zodariidae) that exemplify biomolecular diversity. Journal of Biological Chemistry. 2006;281(30):20983-92.

81. Claeson P, Göransson U, Johansson S, Luijendijk T, Bohlin L. Fractionation protocol for the isolation of polypeptides from plant biomass. Journal of natural products. 1998;61(1):77-81.

82. Göransson U, Luijendijk T, Johansson S, Bohlin L, Claeson P. Seven novel macrocyclic polypeptides from Viola a rvensis. Journal of natural products. 1999;62(2):283-6.

83. Craik DJ, Daly NL, Bond T, Waine C. Plant cyclotides: a unique family of cyclic and knotted proteins that defines the cyclic cystine knot structural motif. Journal of molecular biology. 1999;294(5):1327-36.

84. Herrmann A, Burman R, Mylne JS, Karlsson G, Gullbo J, Craik DJ, et al. The alpine violet, Viola biflora, is a rich source of cyclotides with potent cytotoxicity. Phytochemistry. 2008;69(4):939-52.

85. Stenger S, Hanson DA, Teitelbaum R, Dewan P, Niazi KR, Froelich CJ, et al. An antimicrobial activity of cytolytic T cells mediated by granulysin. Science. 1998;282(5386):121-5.

86. de Lorenzo V. Isolation and characterization of microcin E 492 from Klebsiella pneumoniae. Archives of microbiology. 1984;139:72-5.

87. Conlon JM, Sonnevend A, Davidson C, Smith DD, Nielsen PF. The ascaphins: a family of antimicrobial peptides from the skin secretions of the most primitive extant frog, Ascaphus truei. Biochemical and biophysical research communications. 2004;320(1):170-5.

88. Wang Y, Hong J, Liu X, Yang H, Liu R, Wu J, et al. Snake cathelicidin from Bungarus fasciatus is a potent peptide antibiotics. PloS one. 2008;3(9):e3217.

89. Rohrl J, Yang D, Oppenheim JJ, Hehlgans T. Identification and biological characterization of mouse β-defensin 14, the orthologue of human β-defensin 3. Journal of Biological Chemistry. 2008;283(9):5414-9.

90. Yizeng T, Shuangquan Z, Xianming Q. Separation, purification of antibacterial CM4 and the research of the structure and character. Sci China B. 1989;32:473-80.

91. Samuelsson G, Seger L, Olson T. The amino acid sequence of oxidized viscotoxin A3 from the European mistletoe (Viscum album L, Loranthaceae). Acta Chemica Scandinavica. 1968;22(8):2624-42.

92. Orrù S, Scaloni A, Giannattasio M, Urech K, Pucci P, Schaller G. Amino acid sequence, SS bridge arrangement and distribution in plant tissues of thionins from Viscum album. Biological chemistry. 1997;378(9):989-96.

93. Schrader G, Apel K. Isolation and characterization of cDNAs encoding viscotoxins of mistletoe (Viscum album). European journal of biochemistry. 1991;198(3):549-53.

94. Lee DG, Hahm K-S, Park Y, Kim H-Y, Lee W, Lim S-C, et al. Functional and structural characteristics of anticancer peptide Pep27 analogues. Cancer cell international. 2005;5:1-14.

95. Kong J, Du X, Fan C, Xu J, Zheng X. Determination of primary structure of a novel peptide from mistletoe and its antitumor activity. Yao xue xue bao= Acta Pharmaceutica Sinica. 2004;39(10):813-7.

96. Pan C-Y, Chen J-Y, Cheng Y-SE, Chen C-Y, Ni I-H, Sheen J-F, et al. Gene expression and localization of the epinecidin-1 antimicrobial peptide in the grouper (Epinephelus coioides), and its role in protecting fish against pathogenic infection. DNA and cell biology. 2007;26(6):403-13.

97. Mandal SM, Dey S, Mandal M, Sarkar S, Maria-Neto S, Franco OL. Identification and structural insights of three novel antimicrobial peptides isolated from green coconut water. Peptides. 2009;30(4):633-7.

98. Conlon JM, Ahmed E, Coquet L, Jouenne T, Leprince J, Vaudry H, et al. Peptides with potent cytolytic activity from the skin secretions of the North American leopard frogs, Lithobates blairi and Lithobates yavapaiensis. Toxicon. 2009;53(7-8):699-705.

99. Conlon JM, Demandt A, Nielsen PF, Leprince J, Vaudry H, Woodhams DC. The alyteserins: two families of antimicrobial peptides from the skin secretions of the midwife toad Alytes obstetricans (Alytidae). Peptides. 2009;30(6):1069-73.

100. Čeřovský V, Buděšínský M, Hovorka O, Cvačka J, Voburka Z, Slaninová J, et al. Lasioglossins: three novel antimicrobial peptides from the venom of the eusocial bee Lasioglossum laticeps (Hymenoptera: Halictidae). ChemBioChem. 2009;10(12):2089-99.

101. Li R-F, Wang B, Liu S, Chen S-H, Yu G-H, Yang S-Y, et al. Optimization of the Expression Conditions of CGA-N46 in Bacillus subtilis DB1342 (p-3N46) by Response Surface Methodology. Interdisciplinary Sciences: Computational Life Sciences. 2016;8:277-83.

102. Zhao Z, Ma Y, Dai C, Zhao R, Li S, Wu Y, et al. Imcroporin, a new cationic antimicrobial peptide from the venom of the scorpion Isometrus maculates. Antimicrobial agents and chemotherapy. 2009;53(8):3472-7.

103. Wang C, Sun Z, Liu Y, Zhang X, Xu G. A novel antimicrobial vermipeptide family from earthworm Eisenia fetida. European journal of soil biology. 2007;43:S127-S34.

104. Shamova O, Orlov D, Stegemann C, Czihal P, Hoffmann R, Brogden K, et al. ChBac3. 4: a novel proline-rich antimicrobial peptide from goat leukocytes. International journal of peptide research and therapeutics. 2009;15:31-42.

105. Abbassi F, Lequin O, Piesse C, Goasdoué N, Foulon T, Nicolas P, et al. Temporin-SHf, a new type of phe-rich and hydrophobic ultrashort antimicrobial peptide. Journal of Biological Chemistry. 2010;285(22):16880-92.

106. DIMARCQ JL, Hoffmann D, Meister M, Bulet P, Lanot R, REICHHART JM, et al. Characterization and transcriptional profiles of a Drosophila gene encoding an insect defensin: a study in insect immunity. European Journal of Biochemistry. 1994;221(1):201-9.

107. Lee S-H, Lee D, Yang S-T, Kim Y, Kim J, Hahm K-S, et al. Antibiotic Activity of Reversed Peptides of α-Helical Antimicrobial Peptide, P18. Protein and Peptide Letters. 2002;9(5):395-402.

108. Yount NY, Kupferwasser D, Spisni A, Dutz SM, Ramjan ZH, Sharma S, et al. Selective reciprocity in antimicrobial activity versus cytotoxicity of hBD-2 and crotamine. Proceedings of the National Academy of Sciences. 2009;106(35):14972-7.

109. Nguyen GKT, Zhang S, Nguyen NTK, Nguyen PQT, Chiu MS, Hardjojo A, et al. Discovery and characterization of novel cyclotides originated from chimeric precursors consisting of albumin-1 chain a and cyclotide domains in the Fabaceae family. Journal of Biological Chemistry. 2011;286(27):24275-87.

110. Yeshak MY, Burman R, Asres K, Göransson U. Cyclotides from an extreme habitat: characterization of cyclic peptides from Viola abyssinica of the Ethiopian highlands. Journal of natural products. 2011;74(4):727-31.

111. Mandal SM, Migliolo L, Das S, Mandal M, Franco OL, Hazra TK. Identification and characterization of a bactericidal and proapoptotic peptide from Cycas revoluta seeds with DNA binding properties. Journal of Cellular Biochemistry. 2012;113(1):184-93.

112. He W, Chan LY, Zeng G, Daly NL, Craik DJ, Tan N. Isolation and characterization of cytotoxic cyclotides from Viola philippica. Peptides. 2011;32(8):1719-23.

113. Monincová L, Buděšínský M, Slaninová J, Hovorka O, Cvačka J, Voburka Z, et al. Novel antimicrobial peptides from the venom of the eusocial bee Halictus sexcinctus (Hymenoptera: Halictidae) and their analogs. Amino Acids. 2010;39:763-75.

114. Slaninová J, Mlsová V, Kroupová H, Alán L, Tůmová T, Monincová L, et al. Toxicity study of antimicrobial peptides from wild bee venom and their analogs toward mammalian normal and cancer cells. Peptides. 2012;33(1):18-26.

115. Mechkarska M, Prajeep M, Coquet L, Leprince J, Jouenne T, Vaudry H, et al. The hymenochirins: A family of host-defense peptides from the Congo dwarf clawed frog Hymenochirus boettgeri (Pipidae). Peptides. 2012;35(2):269-75.

116. Zeng X-C, Wang S-X, Zhu Y, Zhu S-Y, Li W-X. Identification and functional characterization of novel scorpion venom peptides with no disulfide bridge from Buthus martensii Karsch. Peptides. 2004;25(2):143-50.

117. Gerlach SL, Burman R, Bohlin L, Mondal D, Goransson U. Isolation, characterization, and bioactivity of cyclotides from the Micronesian plant Psychotria leptothyrsa. Journal of natural products. 2010;73(7):1207-13.

118. Nguyen GKT, Lim WH, Nguyen PQT, Tam JP. Novel cyclotides and uncyclotides with highly shortened precursors from Chassalia chartacea and effects of methionine oxidation on bioactivities. Journal of Biological Chemistry. 2012;287(21):17598-607.

119. Yang D, Chen Q, Hoover DM, Staley P, Tucker KD, Lubkowski J, et al. Many chemokines including CCL20/MIP-3α display antimicrobial activity. Journal of Leucocyte Biology. 2003;74(3):448-55.

120. Conlon JM, Mechkarska M, Coquet L, Jouenne T, Leprince J, Vaudry H, et al. Characterization of antimicrobial peptides in skin secretions from discrete populations of Lithobates chiricahuensis (Ranidae) from central and southern Arizona. Peptides. 2011;32(4):664-9.

121. Peng K-C, Lee S-H, Hour A-L, Pan C-Y, Lee L-H, Chen J-Y. Five different piscidins from Nile tilapia, Oreochromis niloticus: analysis of their expressions and biological functions. PloS one. 2012;7(11):e50263.

122. Ruangsri J, Salger SA, Caipang CM, Kiron V, Fernandes JM. Differential expression and biological activity of two piscidin paralogues and a novel splice variant in Atlantic cod (Gadus morhua L.). Fish & Shellfish Immunology. 2012;32(3):396-406.

123. Zhao R-L, Han J-Y, Han W-Y, He H-X, Ma J-F. Effects of two novel peptides from skin of lithobates catesbeianus on tumor cell morphology and proliferation. Molecular Cloning—Selected Applications in Medicine and Biology. 2011:73-80.

124. Darveau RP, Blake J, Seachord CL, Cosand WL, Cunningham MD, Cassiano-Clough L, et al. Peptides related to the carboxyl terminus of human platelet factor IV with antibacterial activity. The Journal of clinical investigation. 1992;90(2):447-55.

125. Ramírez-Carreto S, Quintero-Hernández V, Jiménez-Vargas JM, Corzo G, Possani LD, Becerril B, et al. Gene cloning and functional characterization of four novel antimicrobial-like peptides from scorpions of the family Vaejovidae. Peptides. 2012;34(2):290-5.

126. Liu Z, Deng M, Xiang J, Ma H, Hu W, Zhao Y, et al. A novel spider peptide toxin suppresses tumor growth through dual signaling pathways. Current molecular medicine. 2012;12(10):1350-60.

127. Guo X, Ma C, Du Q, Wei R, Wang L, Zhou M, et al. Two peptides, TsAP-1 and TsAP-2, from the venom of the Brazilian yellow scorpion, Tityus serrulatus: evaluation of their antimicrobial and anticancer activities. Biochimie. 2013;95(9):1784-94.

128. Dong W, Dong Z, Mao X, Sun Y, Li F, Shang D. Structure-activity analysis and biological studies of chensinin-1b analogues. Acta Biomaterialia. 2016;37:59-68.

129. Li A, Zhang Y, Wang C, Wu G, Wang Z. Purification, molecular cloning, and antimicrobial activity of peptides from the skin secretion of the black-spotted frog, Rana nigromaculata. World Journal of Microbiology and Biotechnology. 2013;29:1941-9.

130. Conlon JM, Prajeep M, Mechkarska M, Coquet L, Leprince J, Jouenne T, et al. Characterization of the host-defense peptides from skin secretions of Merlin's clawed frog Pseudhymenochirus merlini: insights into phylogenetic relationships among the Pipidae. Comparative Biochemistry and Physiology Part D: Genomics and Proteomics. 2013;8(4):352-7.

131. Sen Z, Zhan XK, Jing J, Yi Z, Wanqi Z. Chemosensitizing activities of cyclotides from Clitoria ternatea in paclitaxel-resistant lung cancer cells. Oncology letters. 2013;5(2):641-4.

132. Galvez AF, De Lumen BO. A soybean cDNA encoding a chromatin-binding peptide inhibits mitosis of mammalian cells. Nature biotechnology. 1999;17(5):495-500.

133. Guzmán-Rodríguez JJ, López-Gómez R, Suárez-Rodríguez LM, Salgado-Garciglia R, Rodríguez-Zapata LC, Ochoa-Zarzosa A, et al. Antibacterial activity of defensin PaDef from avocado fruit (Persea americana var. drymifolia) expressed in endothelial cells against Escherichia coli and Staphylococcus aureus. BioMed Research International. 2013;2013(1):986273.

134. Mishra A, Gauri SS, Mukhopadhyay SK, Chatterjee S, Das SS, Mandal SM, et al. Identification and structural characterization of a new pro-apoptotic cyclic octapeptide cyclosaplin from somatic seedlings of Santalum album L. Peptides. 2014;54:148-58.

135. Niggemann J, Bozko P, Bruns N, Wodtke A, Gieseler MT, Thomas K, et al. Baceridin, a cyclic hexapeptide from an epiphytic Bacillus strain, inhibits the proteasome. ChemBioChem. 2014;15(7):1021-9.

136. Conlon JM, Mechkarska M, Radosavljevic G, Attoub S, King JD, Lukic ML, et al. A family of antimicrobial and immunomodulatory peptides related to the frenatins from skin secretions of the Orinoco lime frog Sphaenorhynchus lacteus (Hylidae). Peptides. 2014;56:132-40.

137. Tareq FS, Lee MA, Lee H-S, Lee J-S, Lee Y-J, Shin HJ. Gageostatins A–C, antimicrobial linear lipopeptides from a marine Bacillus subtilis. Marine Drugs. 2014;12(2):871-85.

138. Ghavami S, Asoodeh A, Klonisch T, Halayko AJ, Kadkhoda K, Kroczak TJ, et al. Brevinin‐2R1 semi‐selectively kills cancer cells by a distinct mechanism, which involves the lysosomal‐mitochondrial death pathway. Journal of cellular and molecular medicine. 2008;12(3):1005-22.

139. Falcao CB, de La Torre B, Pérez-Peinado C, Barron AE, Andreu D, Rádis-Baptista G. Vipericidins: a novel family of cathelicidin-related peptides from the venom gland of South American pit vipers. Amino acids. 2014;46:2561-71.

140. Li JinYao LJ, Zhang FuChun ZF, Ma ZhengHai MZ. Prokaryotic expression of cecropin gene isolated from the silkworm Bombyx mori Xinjiang race and antibacterial activity of fusion cecropin. 2004.

141. Du Q, Hou X, Wang L, Zhang Y, Xi X, Wang H, et al. AaeAP1 and AaeAP2: novel antimicrobial peptides from the venom of the scorpion, Androctonus aeneas: structural characterisation, molecular cloning of biosynthetic precursor-encoding cDNAs and engineering of analogues with enhanced antimicrobial and anticancer activities. Toxins. 2015;7(2):219-37.

142. de Melo ET, Estrela AB, Santos ECG, Machado PRL, Farias KJS, Torres TM, et al. Structural characterization of a novel peptide with antimicrobial activity from the venom gland of the scorpion Tityus stigmurus: Stigmurin. Peptides. 2015;68:3-10.

143. Lee JH, Kim I-W, Kim S-H, Kim M, Yun E-Y, Nam S-H, et al. Anticancer activity of the antimicrobial peptide scolopendrasin VII derived from the centipede, Scolopendra subspinipes mutilans. Journal of microbiology and biotechnology. 2015;25(8):1275-80.

144. Esmaeili MA, Abagheri-Mahabadi N, Hashempour H, Farhadpour M, Gruber CW, Ghassempour A. Viola plant cyclotide vigno 5 induces mitochondria-mediated apoptosis via cytochrome C release and caspases activation in cervical cancer cells. Fitoterapia. 2016;109:162-8.

145. Hu E, Wang D, Chen J, Tao X. Novel cyclotides from Hedyotis diffusa induce apoptosis and inhibit proliferation and migration of prostate cancer cells. International journal of clinical and experimental medicine. 2015;8(3):4059.

146. Li S, Hao L, Bao W, Zhang P, Su D, Cheng Y, et al. A novel short anionic antibacterial peptide isolated from the skin of Xenopus laevis with broad antibacterial activity and inhibitory activity against breast cancer cell. Archives of Microbiology. 2016;198:473-82.

147. Theansungnoen T, Maijaroen S, Jangpromma N, Yaraksa N, Daduang S, Temsiripong T, et al. Cationic antimicrobial peptides derived from Crocodylus siamensis leukocyte extract, revealing anticancer activity and apoptotic induction on human cervical cancer cells. The Protein Journal. 2016;35:202-11.

148. Elsayed SS, Trusch F, Deng H, Raab A, Prokes I, Busarakam K, et al. Chaxapeptin, a lasso peptide from extremotolerant Streptomyces leeuwenhoekii strain C58 from the hyperarid Atacama Desert. The Journal of organic chemistry. 2015;80(20):10252-60.

149. Um S, Kim Y-J, Kwon H, Wen H, Kim S-H, Kwon HC, et al. Sungsanpin, a lasso peptide from a deep-sea streptomycete. Journal of Natural Products. 2013;76(5):873-9.

150. Harrison PL, Abdel-Rahman MA, Strong PN, Tawfik MM, Miller K. Characterisation of three alpha-helical antimicrobial peptides from the venom of Scorpio maurus palmatus. Toxicon. 2016;117:30-6.

151. Peel E, Cheng Y, Djordjevic J, Fox S, Sorrell T, Belov K. Cathelicidins in the Tasmanian devil (Sarcophilus harrisii). Scientific reports. 2016;6(1):35019.

152. Jia HP, Wowk SA, Schutte BC, Lee SK, Vivado A, Tack BF, et al. A novel murine β-defensin expressed in tongue, esophagus, and trachea. Journal of Biological Chemistry. 2000;275(43):33314-20.

153. Baindara P, Singh N, Ranjan M, Nallabelli N, Chaudhry V, Pathania GL, et al. Laterosporulin10: a novel defensin like class IId bacteriocin from Brevibacillus sp. strain SKDU10 with inhibitory activity against microbial pathogens. Microbiology. 2016;162(8):1286-99.

154. Peng X, Zhou C, Hou X, Liu Y, Wang Z, Peng X, et al. Molecular characterization and bioactivity evaluation of two novel bombinin peptides from the skin secretion of Oriental fire-bellied toad, Bombina orientalis. Amino Acids. 2018;50:241-53.

155. Huang L, Chen D, Wang L, Lin C, Ma C, Xi X, et al. Dermaseptin-PH: a novel peptide with antimicrobial and anticancer activities from the skin secretion of the South American orange-legged leaf frog, Pithecopus (Phyllomedusa) hypochondrialis. Molecules. 2017;22(10):1805.

156. Xin H, Ji S, Peng J, Han P, An X, Wang S, et al. Isolation and characterisation of a novel antibacterial peptide from a native swine intestinal tract-derived bacterium. International journal of antimicrobial agents. 2017;49(4):427-36.

157. Wu D, Gao Y, Tan Y, Liu Y, Wang L, Zhou M, et al. Discovery of distinctin-like-peptide-PH (DLP-PH) from the skin secretion of Phyllomedusa hypochondrialis, a prototype of a novel family of antimicrobial peptide. Frontiers in microbiology. 2018;9:541.

158. Sang M, Wu Q, Xi X, Ma C, Wang L, Zhou M, et al. Identification and target-modifications of temporin-PE: A novel antimicrobial peptide in the defensive skin secretions of the edible frog, Pelophylax kl. esculentus. Biochemical and Biophysical Research Communications. 2018;495(4):2539-46.

159. Heulot M, Jacquier N, Aeby S, Le Roy D, Roger T, Trofimenko E, et al. The Anticancer Peptide TAT-RasGAP317− 326 Exerts Broad Antimicrobial Activity. Frontiers in microbiology. 2017;8:994.

160. Tan Y, Chen X, Ma C, Xi X, Wang L, Zhou M, et al. Biological activities of cationicity-enhanced and hydrophobicity-optimized analogues of an antimicrobial peptide, dermaseptin-PS3, from the Skin Secretion of Phyllomedusa sauvagii. Toxins. 2018;10(8):320.

161. Zhu H, Ding X, Li W, Lu T, Ma C, Xi X, et al. Discovery of two skin-derived dermaseptins and design of a TAT-fusion analogue with broad-spectrum antimicrobial activity and low cytotoxicity on healthy cells. PeerJ. 2018;6:e5635.

162. Long Q, Li L, Wang H, Li M, Wang L, Zhou M, et al. Novel peptide dermaseptin‐PS 1 exhibits anticancer activity via induction of intrinsic apoptosis signalling. Journal of cellular and molecular medicine. 2019;23(2):1300-12.

163. Chen X, Zhang L, Ma C, Zhang Y, Xi X, Wang L, et al. A novel antimicrobial peptide, Ranatuerin-2PLx, showing therapeutic potential in inhibiting proliferation of cancer cells. Bioscience reports. 2018;38(6):BSR20180710.

164. Chen X, Zhang L, Wu Y, Wang L, Ma C, Xi X, et al. Evaluation of the bioactivity of a mastoparan peptide from wasp venom and of its analogues designed through targeted engineering. International journal of biological sciences. 2018;14(6):599.

165. Notomista E, Falanga A, Fusco S, Pirone L, Zanfardino A, Galdiero S, et al. The identification of a novel Sulfolobus islandicus CAMP-like peptide points to archaeal microorganisms as cell factories for the production of antimicrobial molecules. Microbial cell factories. 2015;14:1-11.

166. Liu J, Wu Q, Li L, Xi X, Wu D, Zhou M, et al. Discovery of phylloseptins that defense against gram-positive bacteria and inhibit the proliferation of the non-small cell lung cancer cell line, from the skin secretions of Phyllomedusa frogs. Molecules. 2017;22(9):1428.

167. Anju A, Smitha C, Preetha K, Boobal R, Rosamma P. Molecular characterization, recombinant expression and bioactivity profile of an antimicrobial peptide, Ss-arasin from the Indian mud crab, Scylla serrata. Fish & Shellfish Immunology. 2019;88:352-8.

168. Li B, Lyu P, Xie S, Qin H, Pu W, Xu H, et al. LFB: a novel antimicrobial brevinin-like peptide from the skin secretion of the Fujian large headed frog, Limnonectes fujianensi. Biomolecules. 2019;9(6):242.

169. Li M, Xi X, Ma C, Chen X, Zhou M, Burrows JF, et al. A novel dermaseptin isolated from the skin secretion of phyllomedusa tarsius and its cationicity-enhanced analogue exhibiting effective antimicrobial and anti-proliferative activities. Biomolecules. 2019;9(10):628.

170. Hansen IK, Isaksson J, Poth AG, Hansen KØ, Andersen AJ, Richard CS, et al. Isolation and characterization of antimicrobial peptides with unusual disulfide connectivity from the colonial ascidian Synoicum turgens. Marine drugs. 2020;18(1):51.

171. Santana CJC, Magalhães ACM, Prías-Márquez CA, Falico DA, dos Santos Júnior AC, Lima BD, et al. Biological properties of a novel multifunctional host defense peptide from the skin secretion of the Chaco tree frog, Boana raniceps. Biomolecules. 2020;10(5):790.

172. Wu Y, Wang L, Zhou M, Chen T, Shaw C. Phylloseptin-PBa1,-PBa2,-PBa3: Three novel antimicrobial peptides from the skin secretion of Burmeister's leaf frog (Phyllomedusa burmeisteri). Biochemical and biophysical research communications. 2019;509(3):664-73.

173. Wang H, He H, Chen X, Zhou M, Wei M, Xi X, et al. A novel antimicrobial peptide (Kassinatuerin-3) isolated from the skin secretion of the African frog, Kassina senegalensis. Biology. 2020;9(7):148.

174. Shi D, Hou X, Wang L, Gao Y, Wu D, Xi X, et al. Two novel dermaseptin-like antimicrobial peptides with anticancer activities from the skin secretion of Pachymedusa dacnicolor. Toxins. 2016;8(5):144.

175. Morishima I, Suginaka S, Ueno T, Hirano H. Isolation and structure of cecropins, inducible antibacterial peptides, from the silkworm, Bombyx mori. Comparative biochemistry and physiology B, Comparative biochemistry. 1990;95(3):551-4.

176. Santana CJC, Magalhães ACM, dos Santos Júnior AC, Ricart CAO, Lima BD, Álvares AdCM, et al. Figainin 1, a novel amphibian skin peptide with antimicrobial and antiproliferative properties. Antibiotics. 2020;9(9):625.

177. Lugo JM, Tafalla C, Oliva A, Pons T, Oliva B, Aquilino C, et al. Evidence for antimicrobial and anticancer activity of pituitary adenylate cyclase-activating polypeptide (PACAP) from North African catfish (Clarias gariepinus): Its potential use as novel therapeutic agent in fish and humans. Fish & shellfish immunology. 2019;86:559-70.

178. Vineeth Kumar T, Asha R, George S. Identification and functional characterisation of Esculentin-2 HYba peptides and their C-terminally amidated analogs from the skin secretion of an endemic frog. Natural product research. 2021;35(8):1262-6.

179. Gopalakrishnan S, Uma SK, Mohan G, Mohan A, Shanmugam G, Kumar VT, et al. SSTP1, a host defense peptide, exploits the immunomodulatory il6 pathway to induce apoptosis in cancer cells. Frontiers in Immunology. 2021;12:740620.

180. Qin H, Fang H, Chen X, Wang L, Ma C, Xi X, et al. Exploration of the structure–function relationships of a novel frog skin secretion-derived bioactive peptide, t-DPH1, through use of rational design, cationicity enhancement and in vitro studies. Antibiotics. 2021;10(12):1529.

181. Gao Y, Chai J, Wu J, Zeng Q, Guo R, Chen X, et al. Molecular cloning and characterization of a novel antimicrobial peptide from the skin of Kaloula pulchra. Current Pharmaceutical Biotechnology. 2022;23(15):1873-82.

182. Lu C, Liu L, Ma C, Di L, Chen T. A novel antimicrobial peptide found in Pelophylax nigromaculatus. Journal of Genetic Engineering and Biotechnology. 2022;20(1):76.

183. Conlon JM, Hunter L, Attoub S, Casciaro B, Mechkarska M, Abdel‐Wahab YH. Antimicrobial, cytotoxic, and insulin‐releasing activities of the amphibian host‐defense peptide ocellatin‐3N and its L‐lysine‐substituted analogs. Journal of Peptide Science. 2023;29(4):e3463.

184. de Moraes LFRN, Silva PSe, Pereira TCPL, Almeida Rodrigues TA, Farias Frihling BE, da Costa RA, et al. First generation of multifunctional peptides derived from latarcin-3a from Lachesana tarabaevi spider toxin. Frontiers in Microbiology. 2022;13:965621.

185. Rogozhin EA, Sadykova VS, Baranova AA, Vasilchenko AS, Lushpa VA, Mineev KS, et al. A novel lipopeptaibol emericellipsin A with antimicrobial and antitumor activity produced by the extremophilic fungus Emericellopsis alkalina. Molecules. 2018;23(11):2785.

186. Freitas GGd, Barbosa JM, Santana CJCd, Magalhães ACM, Macedo KWR, Souza JOd, et al. Purification and biological properties of raniseptins-3 and-6, two antimicrobial peptides from Boana raniceps (Cope, 1862) skin secretion. Biomolecules. 2023;13(3):576.

187. Pestonjamasp VK, Huttner KH, Gallo RL. Processing site and gene structure for the murine antimicrobial peptide CRAMP. Peptides. 2001;22(10):1643-50.

188. Shin SY, Kang S-W, Lee DG, Eom SH, Song WK, Kim JI. CRAMP analogues having potent antibiotic activity against bacterial, fungal, and tumor cells without hemolytic activity. Biochemical and Biophysical Research Communications. 2000;275(3):904-9.

189. Qin H, Zuo W, Ge L, Siu SW, Wang L, Chen X, et al. Discovery and analysis of a novel antimicrobial peptide B1AW from the skin secretion of Amolops wuyiensis and improving the membrane-binding affinity through the construction of the lysine-introduced analogue. Computational and Structural Biotechnology Journal. 2023;21:2960-72.

190. Conlon JM, Guilhaudis L, Attoub S, Coquet L, Leprince J, Jouenne T, et al. Purification, Conformational Analysis and Cytotoxic Activities of Host-Defense Peptides from the Giant Gladiator Treefrog Boana boans (Hylidae: Hylinae). Antibiotics. 2023;12(7):1102.

191. Ma X, Chen Y, Shu A, Jiang Y, Chen X, Ma C, et al. A novel antimicrobial peptide, Dermaseptin-SS1, with anti-proliferative activity, isolated from the skin secretion of Phyllomedusa tarsius. Molecules. 2023;28(18):6558.

192. Thi Phuong HB, Huy BL, Van KN, Thi ND, Thi TB, Thi Hai YN, et al. Reducing Self-Assembly by Increasing Net Charge: Effect on Biological Activity of Mastoparan C. ACS Medicinal Chemistry Letters. 2023;15(1):69-75.

193. Yao A, Liu T, Cai Y, Zhou S, Chen X, Zhou M, et al. Progressive Design of a Ranatuerin-2 Peptide from Amolops wuyiensis: Enhancement of Bioactivity and In Vivo Efficacy. Antibiotics. 2023;13(1):5.

194. Conlon JM, Sridhar A, Khan D, Cunning TS, Delaney JJ, Taggart MG, et al. Multifunctional host-defense peptides isolated from skin secretions of the banana tree dwelling frog Boana platanera (Hylidae; Hylinae). Biochimie. 2024;223:23-30.

195. Chen Z, Wang L, He D, Liu Q, Han Q, Zhang J, et al. Exploration of the Antibacterial and Anti-Inflammatory Activity of a Novel Antimicrobial Peptide Brevinin-1BW. Molecules. 2024;29(7):1534.

196. Parente AM, Daniele-Silva A, Furtado AA, Melo MA, Lacerda AF, Queiroz M, et al. Analogs of the scorpion venom peptide stigmurin: structural assessment, toxicity, and increased antimicrobial activity. Toxins. 2018;10(4):161.

197. Chen D, Zhou X, Chen X, Huang L, Xi X, Ma C, et al. Evaluating the bioactivity of a novel antimicrobial and anticancer peptide, dermaseptin-PS4 (Der-PS4), from the skin secretion of Phyllomedusa sauvagii. Molecules. 2019;24(16):2974.

198. Zhong K, Wang Y, Wang Z, Zhang Z, Zhao S, Li H, et al. AP-64, encoded by C5orf46, exhibits antimicrobial activity against gram-negative bacteria. Biomolecules. 2021;11(4):485.
